# Genetic Landscape of Relapsed and Refractory Diffuse Large B-Cell Lymphoma: A Systemic Review and Association Analysis With Next-Generation Sequencing

**DOI:** 10.3389/fgene.2021.677650

**Published:** 2021-12-02

**Authors:** Fan Gao, Lei Tian, Hui Shi, Peihao Zheng, Jing Wang, Fei Dong, Kai Hu, Xiaoyan Ke

**Affiliations:** ^1^ Department of Hematology, Peking University Third Hospital, Beijing, China; ^2^ Department of Adult Lymphoma, Beijing Boren Hospital, Beijing, China

**Keywords:** diffuse large-cell lymphoma, whole exome sequencing (WES), suppressor of cytokine signaling 1 protein (SOCS1), STAT6 transcription factor, ITPKB protein

## Abstract

In our research, we screened 1,495 documents, compiled the whole-exome sequencing data of several studies, formed a data set including 92 observations of RRDLBCL (Relapsed and refractory diffuse large B-cell lymphoma), and performed association analysis on the high-frequency mutations among them. The most common mutations in the data set include TTN, KMT2D, TP53, IGLL5, CREBBP, BCL2, MYD88, and SOCS1 etc. Among these, CREBBP, KMT2D, and BCL2 have a strong association with each other, and SOCS1 has a strong association with genes such as STAT6, ACTB, CIITA, ITPKB, and GNA13. TP53 lacks significant associations with most genes. Through SOM clustering, expression-level analysis and protein interaction analysis of common gene mutations, we believe that RRDLBCL can be divided into five main types. We tested the function of the model and described the clinical characteristics of each subtype through a targeted sequencing RRDLBCL cohort of 96 patients. The classification is stated as follows: 1) JAK-STAT-related type: including STAT6, SOCS1, CIITA, etc. The genetic lineage is similar to PMBL and cHL. Retrospective analysis suggests that this subtype responds poorly to induction therapy (R-CHOP, *p* < 0.05). 2) BCL-CREBBP type: Epigenetic mutations such as KMT2D and CREBBP are more common in this type, and are often accompanied by BCL2 and EZH2 mutations. 3) MCD type: including MYD88 and CD79B, PIM1 is more common in this subtype. 4) TP53 mutation: TP53 mutant patients, which suggests the worst prognosis (*p* < 0.05) and worst response to CART treatment. 5) Undefined type (Sparse item type): Major Genetic Change Lacking Type, which has a better prognosis and better response to CART treatment. We also reviewed the literature from recent years concerning the previously mentioned common gene mutations.

## 1 Introduction

Diffuse large B-cell lymphoma (DLBCL) is the most common histologic subtype of non-Hodgkin lymphoma (NHL). For patients who are refractory to initial treatment or relapse after an initial response, only a small percentage will experience prolonged disease-free survival with salvage chemoimmunotherapy alone (Gisselbrecht et al., 2010). Recent research results have shown that for patients with aggressive B-cell lymphoma within 12 months after autologous stem cell transplantation and resistant to chemotherapy, the objective response rate of combined chemoimmunotherapy is 26%, and the complete response rate is 7%. The average overall survival time is only 6.3 months (Philip et al., 1995). These patients and those who are not suitable for hematopoietic stem cell transplantation may become candidates for chimeric antigen receptor T cell therapy (CART therapy) and other targeted therapies. However, due to the significant heterogeneity of DLBCL, the opportunity for physicians to try treatment in these patients is very valuable. How to choose individualized treatment suitable for patients is still a major challenge for hematologists.

In recent years, our understanding of the classification of diffuse large B lymphomas has deepened, and these advances have also been applied to the study of relapsed and refractory diffuse large B-cell lymphomas (RRDLBCL). Among recent advances, the multi-platform genome analysis performed by Schmitz et al. (2018), based on gene expression classification of DLBCL, adds genetic classification that may be helpful for understanding the pathogenesis of DLBCL. This milestone study identified four prominent genetic subtypes in DLBCL as MCD, BN2, N1 and EZB. Recently, Sha et al. (2019) defined a class of molecular high-grade diffuse large B-cell lymphoma (MHG) in their research and concluded that different treatment strategies were required for this group of patients. Chapuy et al. (2018) used the Consensus clustering method to integrate the genetic drivers of DLBCL and identified five subsets, namely C0-C5. More recently, Lacy et al. (2020) applied bernoulli mixture model clustering and analyzed five molecular subtypes of DLBCL based on a cohort of 928 cases of DLBCL, namely MYD88, BCL2, SOCS1/SGK1, TET2/SGK1, and NOTCH2, and an unclassified group. In the same period, Wright et al. (2020) Wright GW published a probabilistic classification tool, which used a similar algorithm to Schmitz and added two subtypes, ST2 and A53. With the contributions of these researchers, our understanding of the heterogeneity of DLBCL has reached a critical point. Since DLBCL includes various genetic subtypes, it is necessary to understand what role these components play in relapsed and refractory DLBCL. In the study by Schmitz, the results of survival analysis showed that MCD and N1 have the worst prognosis in the activated B-cell (ABC) subtype, while EZB has the worst prognosis in the germinal center B-cell (GCB) subtype. As clinicians, we pay particular attention to the composition of each genetic subtype in RRDLBCL. At the same time, whether there are undisclosed subtypes remains unclear. Further findings in this area can guide clinical design of new treatment strategies to improve the prognosis of patients with RRDLBCL. At the same time, we are also very concerned about whether there are similar genetic clusters in the Chinese population.

At present, there are not enough large-scale studies to summarize the genetic characteristics of this particular population of RRDLBCL patients. To address this, we screened 1,495 documents at first, from which we summarized whole-exome sequencing data from 4 studies of RRDLBCL (Mareschal et al., 2016; Morin et al., 2016; Park et al., 2016; Greenawalt et al., 2017). By conducting an association study of whole-exome sequencing results for a total of 92 patients, we observed the most common mutations in RRDLBCL and attempted to cluster them. In addition, for the most high-frequency genes, we also used another data set (GSE10846) (Lenz et al., 2008) to study the correlation between expression levels, and protein interaction network was also done. Based on the model established with the research of public data, we further analyzed its significance in RRDLBCL through the targeted sequencing results of a cooperative cohort of 96 RRDLBCL patients, and the clinical differences of different genetic subtypes of RRDLBCL were evaluated at the same time. Finally, we reviewed the research progress on these genes, and speculated the possible molecular biological mechanism of RRDLBCL.

## 2 Materials and Methods

### 2.1 Study Selection

A systematic literature search for all relevant articles from January 2005 through August 2019 was conducted in MEDLINE. The search strategy combined the Medical Subject Headings and key words with terms for diffuse large B-cell lymphoma (DLBCL) and for whole-exome sequencing (WES). No language restrictions were imposed. Two investigators (T.L. and W.J.) reviewed all potentially relevant articles independently. We included both prospective and retrospective studies and, critically, complete whole-exome sequencing results of RRDLBCL. One reviewer (T.L.) extracted whole-exome sequencing data and clinical information from each eligible study, and another reviewer (W.J.) confirmed the data. In the end, we selected four articles from 1,495 articles (Table 1) (Mareschal et al., 2016; Morin et al., 2016; Park et al., 2016; Greenawalt et al., 2017), and the data came from five independent clinical studies. We extracted the following information from eligible studies: first author, year of publication, journal, pathological subtypes of RRDLBCL, and whole-exome sequencing results (mutated genes, mutation types, etc.) corresponding to RRDLBCL patients.

**TABLE 1:** List of all selected articles.

Author	Year^a^	Title	Number^b^	Included subtypes	Identifier	WES preparation
Mareschal et al.	2016	Whole-exome sequencing of relapsed/refractory patients expands the repertoire of somatic mutations in diffuse large B-cell lymphoma	14	GCB, ABC, PMBL	GHE	SureSelect Human All Exon V5 kit (Agilent Technologies, Santa Clara, CA, United States)
Greenawalt et al.	2017	Comparative analysis of primary versus relapse/refractory DLBCL identifies shifts in mutation spectrum	47	GCB, ABC, unclassified	E	NimbleGen Sequence Capture Human Exome 2.1M Array (Roche NimbleGen, Inc.)/Agilent SureSelect
Park et al.	2016	Whole-exome and transcriptome sequencing of refractory diffuse large B-cell lymphoma	6	GCB, non-GCB	F	SureSelect Human All Exon 50M (Agilent Technologies, Santa Clara, CA, United States)
Morin et al.	2016	Genetic landscapes of relapsed and refractory diffuse large B-cell lymphomas	25	GCB, ABC, unclassified (NA)	QC-2/CH	Not mentioned; sequenced with the HiSeq 2000 platform (Illumina)

Abbreviation: ABC, activated B cell-like; GCB, germinal center B cell-like; PMBL, primary mediastinal large B cell lymphoma.

a Year: the year of publication.

b Number: number of RRDLBCL patients.

### 2.2 Data Processing and Analysis

We standardized whole-exome sequencing extracted from different studies (standardization of mutation-type tags, Supplementary Material S1) and adopted exclusion criteria to avoid serious bias: 1) Divided the dataset into two groups of similar scale, one of which was the largest independent study (ID starts with E, 47 cases), and the other group included the remaining 45 patients; 2) Excluded genes with significant differences in frequency between the two groups (Fisher’s Exact Test, *p* < 0.001); 3) Excluded gene mutations with frequency less than 0.03 in the whole dataset and the larger group; 4) Mutation type labels adopted: Truncating SNV, Splice site SNV, Missense SNV, Indel (insert or delete), Synonymous SNV, Multiple types (refers to the presence of multiple types of mutations in the same patient sample); 4) Used consistent gene symbols according to ENSEMBL (GRCh37). After the above processing, we obtained a data set consisting of 92 observations and 256 variables (genes). All mutations were confirmed in the literatures as somatic mutations.

We then conducted an association analysis [Apriori and self-organizing map (SOM) algorithms, both based on the R package] on the data set to analyze the strength of the association between each gene and try to cluster it. For genes with strong associations, we rely on another data set (GSE10846) to analyze the correlation of their expressions with of R package (Linear Regression and Pearson Correlation Coefficient). Protein interaction analysis based on String database (Version 11.5). For the specific method, please refer to Supplemental Material SI. Visualization of relevant results was achieved using the R package (Version 4.1.0) and Gephi 0.9.2. Log-Rank test is used for survival analysis, and Student t test is used to compare subgroups of different gene expression levels. Chi-square test is used to test the difference in therapeutic efficacy of different subtypes.

### 2.3 Method of Targeted Sequencing Analysis

**FIGURE 1:**
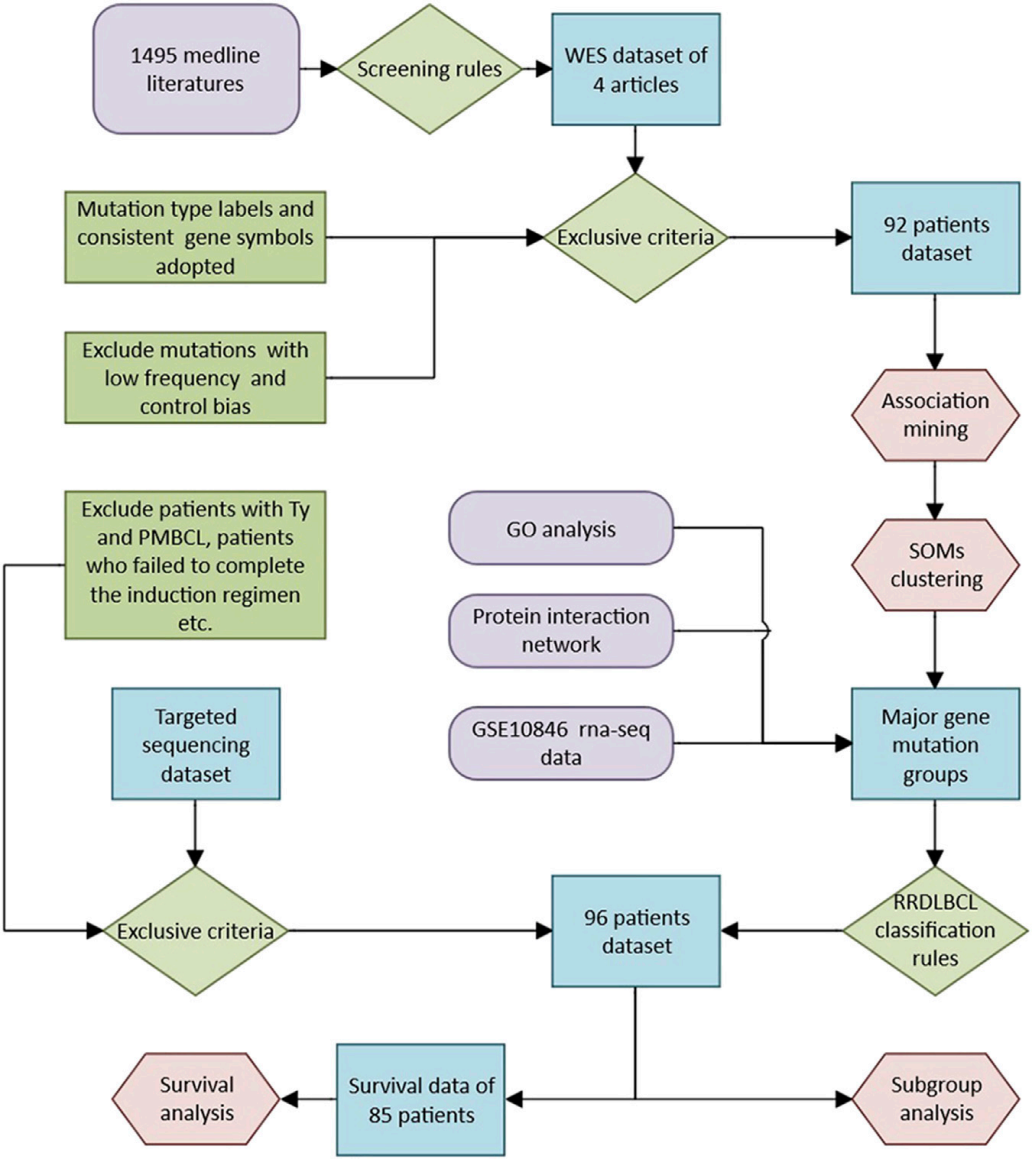
Roadmap of the research method of this study.

We built a model based on the research of public data, and further analyzed its significance in RRDLBCL through the results of targeted sequencing. The data of the targeted sequencing retrospective study came from a collaborative cohort between Peking University Third Hospital and Beijing Boren Hospital. The original cohort included patients with refractory and relapsed DLBCL and PMBCL, and was reviewed by the ethics committee and obtained approval. In this study, the following criteria were used to further screen objects: The selection criteria were: 1) All patients were diagnosed as DLBCL lymphoma by the Department of Pathology, Peking University Third Hospital on newly diagnosed biopsy specimens; 2) Patients were 18 years or older; 3) Two investigators (G.F and S.H) review the medical history to confirm that it is RRDLBCL: relapsed DLBCL refers to the condition of relapse after initial treatment has achieved complete response (Complete response, CR); refractory DLBCL refers to patients who have received standard induction regimen (R-CHOP regimen) for at least 4 courses and found that the clinical remission of lymph nodes and/or involved organs is incomplete [Partial remission (PR) is not achieved], or new lesions appear during treatment. The evaluation criteria meet the 2014 lugano criteria (Cheson et al., 2014). The exclusion criteria were: 1) patients with Tly (transforming lymphoma) and PMBCL (mediastinal large B-cell lymphoma); 2) patients who failed to complete the induction regimen due to reasons other than disease progression; 3) if they were recurring In DLBCL cases, the samples used for sequencing in the included cases must be derived from the biopsy results of recurrence, and the results of the first diagnosis will be excluded. A core panel of 339 genes selected on the basis of prior implication in the pathogenesis of hematologic disease was analyzed in R&R DLBCL disease. The complete set of biotinylated long oligonucleotide probes provided by Roche NimbleGen to perform sequence capture of 339 genes (all coding exons). For the specific method, please refer to Supplemental Material SI. The results of targeted sequencing can be seen in Supplementary Material SIII, which includes evaluation for the potential impact of a variant on the function of a gene [based on CADD, variants with low CADD scores (<10) was removed]. The complete method route is shown in Figure 1.

## 3 Results

### 3.1 Association Mining

**FIGURE 2:**
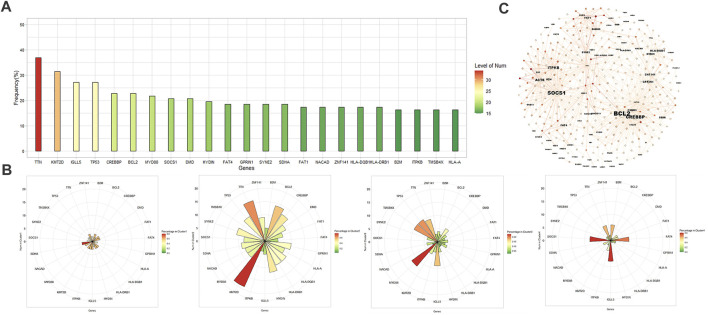
**(A)** Histogram of frequent exon mutant genes in 92 patients. **(B)** From left to right: clusters 1–4 obtained by K-means clustering and their major mutations, and colors of charts represent the percentage of patients with a certain gene mutation in cluster. **(C)** Visualization of 300 rules with Gephi (Fruchterman Reingold layout). The shade depends on the lift value, and label size is proportional to the in-degree, the number of rules the consequent participates in.

The whole exome sequencing data set of 92 patients processed by the aforementioned method is relatively sparse. In this data set, the most common mutations are in TTN (34/92, 37.0%), KMT2D (29/92, 31.5%), TP53 (25/92, 27.2%), IGLL5 (25/92, 27.2%), CREBBP (21/92, 22.8%), BCL2 (21/92, 22.8%), MYD88 (20/92, 21.7%), and SOCS1 (19/92, 20.7%). These genes are also frequent item sets in association analysis (Figure 2A). After K-means clustering the data set, the data was divided into four groups (Figure 2B; Supplementary Figure S1A). Cluster1 was too short and lacked of meaning. Cluster 2 consisted mainly of patients with KMT2D mutations. The most common mutations in the group were KMT2D (19/25, 76%), TTN (16/25, 64%), BCL2 (14/25, 56%) and CREBBP (12/25, 48%). The most common mutations in cluster 3 were MYD88 (12/52, 23.1%). The most common mutations in cluster 4 were IGLL5 (8/10, 80%) and SOCS1 (8/10, 80%). Only 1 of 52 patients in cluster 3 had SOCS1 mutations. Correspondingly, no one had MYD88 mutations in cluster4. Notably, in the whole data set, only one patient has both MYD88 and SOCS1 mutation, only three patients have both MYD88 and CREBBP mutation, and the number of patients with TP53, MYD88, and SOCS1 mutation is 0 (Supplementary Figure S2).

We used the apriori algorithm to observe the association between genes, and found that CREBBP, KMT2D, and BCL2 have a strong association with each other, and SOCS1 has a strong association with STAT6, ACTB, CIITA, ITPKB, GNA13 (lift = 2.77∼3.85), which means that these genes often appear in the form of co-mutation (Supplementary Figures S1B,C). Among patients with SOCS1 mutations, 42.1% (8/19) also have STAT6 mutations (lift = 2.85). TP53 and MYD88 lack a meaningful association with most high frequency genes (lift ≤ 1 indicates independence) (Supplementary Figures S1D,E). Among genes with a relatively high incidence (*n* ≥ 10), MYD88 is most strongly associated with PIM1 and HLA-A (lift of 1.88 and 2.34). Figure 2C shows the 300 rules with the highest lift value in the apriori algorithm, displayed based on the Fruchterman-Reingold layout. Genes such as SOCS1, BCL2, KMT2D, and CREBBP have more association rules with higher lift values, so the level of in-degree is higher. Although the incidence of TTN, IGLL5, DMD, etc is also high, their strength of associations with other genes is relatively weak.

### 3.2 Gene Clustering Based on SOM Self-Organizing Mapping Neural Network Model

In order to further group the gene mutations in RRDLBCL, we took the lift of genes in the dataset relative to SOCS1, KMT2D, TP53 and MYD88 as variables, and clustered them by SOM algorithm. Supplementary Figure S3A shows the results of clustering, where the size of the slice reflects the influence of variables on the objects in the cluster. The mean distance of objects to their closest code book vectors is 0.999, which indicates good mapping quality. By evaluating the generated SOM clusters, a set of ideal clusters were produced (Supplementary Figure S3B from left to right and from bottom to top, clusters 1–16 respectively). Evaluation shows that all clusters are ideal.

**FIGURE 3:**
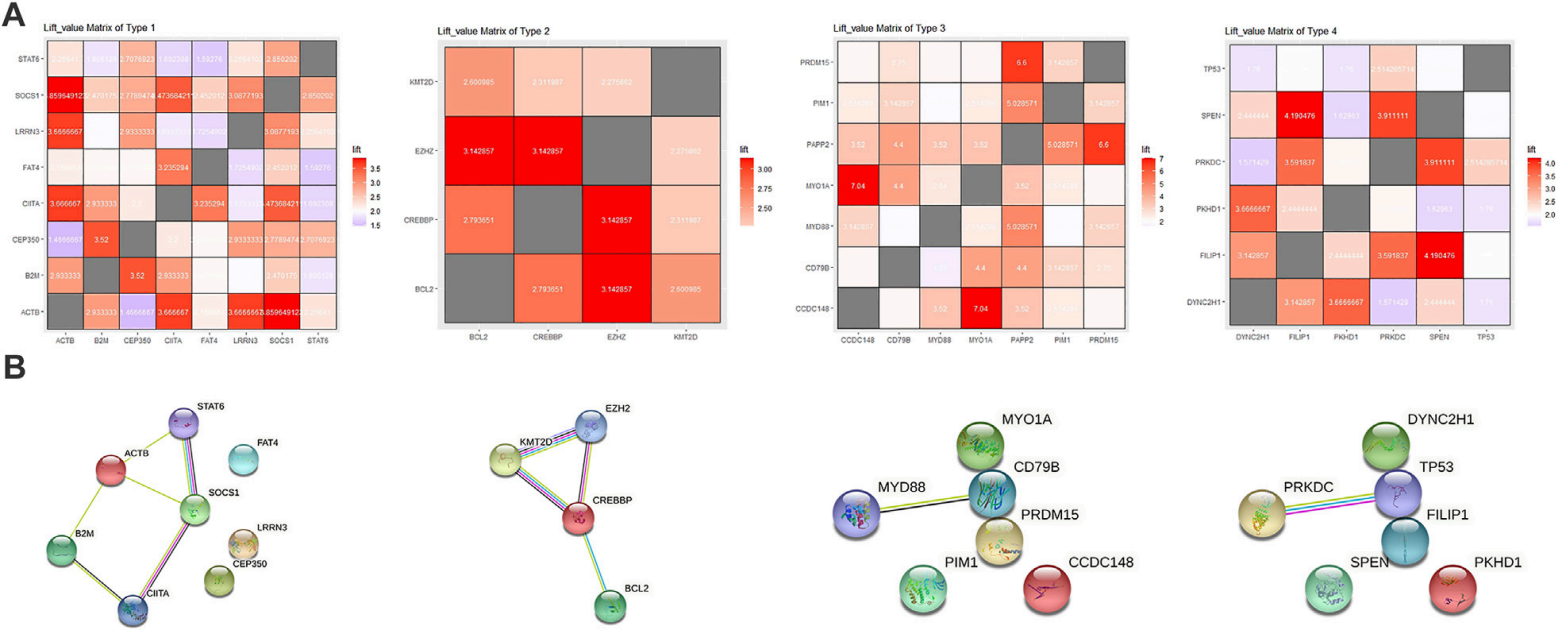
**(A)** From left to right: Matrix heat map of lift values between genes in Type 1–4, and colors represent lift value. **(B)** From left to right: The protein interaction network of Type 1–4 mapped with the string online tool.

Through cluster analysis based on a SOM self-organizing network, we found that one cluster was a group of mutations including SOCS1 and STAT6 (cluster 4, also includes ITPKB, and ACTB), cluster 3 (includes FAT4, MAGI2 etc.) and cluster 8 (includes B2M, CIITA etc.) also showed strong association with the SOCS1 and may be related to the JAK-STAT pathway. We merged these three clusters as Type 1. Cluster 16 was a group of genes, including BCL2, CREBBP and EZH2, related to epigenetic changes and strongly associated with KMT2D. We took this cluster as Type2. High-frequency genes such as MYD88 and TP53 are relatively independent. Cluster 13 (includes BCOR, TEX11 etc.) and cluster 10 (includes PIM1, PRDM15, CD79B etc.) showed strong association with MYD88 relatively, which were merged as Type 3. cluster 12 was a cluster of genes associated with TP53, including SPEN, PRKDC and ANK2 etc., which was taken as Type4 (Figure 3A).

### 3.3 Analysis of Correlation Between Genes by Expression Array Data, GO Enrichment Analysis and Protein Interaction Network

In order to further analyze the relationship between hot-spot genes in RRDLBCL revealed by whole-exome sequencing, we used another public dataset based on whole human genome expression array of Affymetrix Human Genome U133 Plus 2.0 Array. Based on the framework provided by SOM self-organizing network clustering analysis, we perform linear analysis between the expression data of hot-spot genes (Supplementary Figure S4). We found that STAT6 and SOCS1 not only showed a strong correlation in association analysis, but their expression levels were also highly correlated (*p* < 0.0001). In Type 1, the expression levels of 36.7% of the included genes was linearly correlated with SOCS1 and STAT6 (*p* < 0.05), which were selected for further analysis. Similarly, most genes (7/10, 70%) in Type 2 were linearly correlated to CREBBP at the expression level (*p* > 0.05), and were selected for further analysis. And in Type 3, most genes were correlated to PIM1 (12/17, 70.6%, *p* < 0.01). In Type 4, TP53 played the role, which were correlated to 63.6% genes in the group (7/11, *p* < 0.05). We retained genes that had relatively strong associations (lift > 1.5) between each pair in the groups and performed GO enrichment analysis on the selected genes of each type. GO enrichment analysis revealed that Type 1 involved interferon-gamma-mediated signaling pathway, and Type 2 involved beta-catenin-TCF complex and histone methylation. Type 3 involved Toll-like receptor binding, and Type 4 involved regulation of cellular senescence and signal transduction with G1 DNA damage checkpoint (Supplementary Figure S5).

**FIGURE 4:**
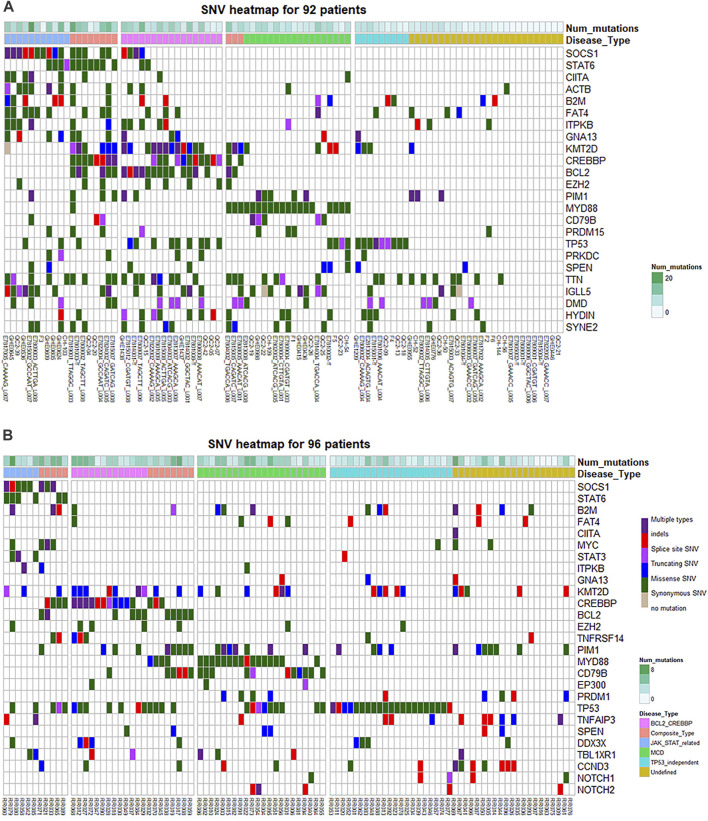
Heat map of gene mutations of different subtypes. **(A)** Mutations of 92 patients in the cohort of public data. **(B)** Mutations of 96 RRDLBCL patients in the Chinese NGS cohort. “Num mutations” refers to the number of gene mutation types carried by a patient. TP53_independent TP53-independent type refers to TP53 mutant patients without genetic characteristics such as SOCS1-STAT6, BCL-CREBBP, and MYD88-CD79B.

Through protein interaction analysis, we found that in Type 1, SOCS1, STAT6 and CIITA interacted closely, which also interacted with B2M and ACTB at the same time. In Type 2, CREBBP, KMT2D and EZH2 interacted closely, and BCL2 was related to CREBBP as well. In Type 3 and Type 4, only two combinations were extracted, which were MYD88-CD79B and TP53-PRKDC (Figure 3B). Through the above analysis, we believe that there may be several combinations in RRDLBCL that summarize most of the mutation types: 1) mutations represented by STAT6 and SOCS1 that may be related to the JAK-STAT pathway; 2) KMT2D, CREBBP, and BCL2 mutations; 3) MYD88, CD79B, and other mutations that show strong correlation with PIM1; 4) TP53 mutations, some of which are driving RRDLBCL independently. Based on this classification, we classified 92 patients (Supplementary Material SI) to observe the situation of each sub-category through Figure 4A; patients on the left side of the heat map mainly carry SOCS1 and STAT6 mutations, and also include mutations considered to be closely related to SOCS1 and STAT6, such as ITPKB, CIITA, FAT4, and B2M. In the middle of the heat map, there are mainly KMT2D, CREBBP, and BCL2 mutations. These three mutations are concentrated in some RRDLBCL patients. To the right of the BCL2-CREBBP patient group on the heat map, there are patients with MYD88 or CD79B mutations, and about 1/4 (6/23) of them also carry PIM1 mutations. However, PIM1 mutations also appear in both JAK-STAT6-related type and BCL2-CREBBP. There is some overlap between these categories, and we call them complex types of RRDLBCL patients.

### 3.4 Targeted Sequencing Results and Subgroup Analysis, Survival Analysis

In the targeted sequencing cohort, a total of 96 patients were included, and a total of 85 patients had complete follow-up data. At first, we used a new data set to verify the reproducibility of the association between the genetic mutations summarized above. We still use the apriori package with the same parameters. Among the 96 patients with targeted sequencing, SOCS1 and STAT6 still showed a strong correlation (lift = 5.93). BCL2 has a strong association with CREBBP in targeted sequencing data (lift = 1.9), but a relatively weak association with KMT2D (lift = 1.46). There are also meaningful association rules between CREBBP and KMT2D (lift = 1.70). MYD88 is still strongly associated with CD79B (lift = 1.67), but is weakly associated with PIM1 (lift = 1.23). TP53 is only strongly associated with DDX3X (lift = 1.58). Although TP53 occupies a high proportion (40/96, 41.6%) in this data set, it still shows obvious independence in the association analysis.

Based on the above analysis results, we relied on the following grouping methods to group cases: 1) JAK-STAT related group: carrying SOCS1, STAT6 mutations; 2) BCL2-CREBBP group: carrying BCL2 or CREBBP mutations; 3) MCD group: Carry MYD88 or CD79B mutations; 4) TP53 mutation group: TP53 mutant patients. According to this classification, there are 11 cases in the JAK-STAT related group (11/96, 11.5%; 9/85, 10.6%); 22 cases in the BCL-CREBBP group (22/96, 22.9%; 21/85, 24.7%); 31 cases in the MCD group (31/96, 32.3%; 27/85, 31.8%); 40 cases in the TP53 group (40/96, 41.6%; 36/85, 42.3%); cases not included in the above groups were classified into the Sparse-itemset group (Undefined), a total of 25 cases (25/96, 26.0%; 23/85, 27.1%). Among them, 66.6% of JAK-STAT related types are GCB (6/9, 66.6%); BCL-CREBBP type is also mainly GCB type (14/21, 66.6%); MCD type is mainly Non-GCB (22/27, 81.5%); TP53 type (22/36, 61.1%) and Sparse-item are also mainly Non-GCB (20/23, 87.0%). However, through the Chi-square test evaluation, only BCL-CREBBP, MCD and Sparse-item had significant difference in the distribution of CCO pathological subtypes (*p* < 0.05). In addition to SOCS1 and STAT6, the main mutations of JAK-STAT-related types include MYC (4/11, 36.4%), B2M (4/11, 36.4%) and STAT3 (3/11, 27.3%). TNFRSF14 mainly appeared in BCL2-CREBBP group (5/23, 21.7%); CCND3 (8/25, 32.0%) and TNFAIP3 (6/25, 24.0%) mainly appeared in Sparse-item group (Supplementary Table S2). The differences in the distribution of the above genes were statistically significant (*p* < 0.05). The gene heat map drawn according to the above grouping is shown in Figure 4B.

**FIGURE 5:**
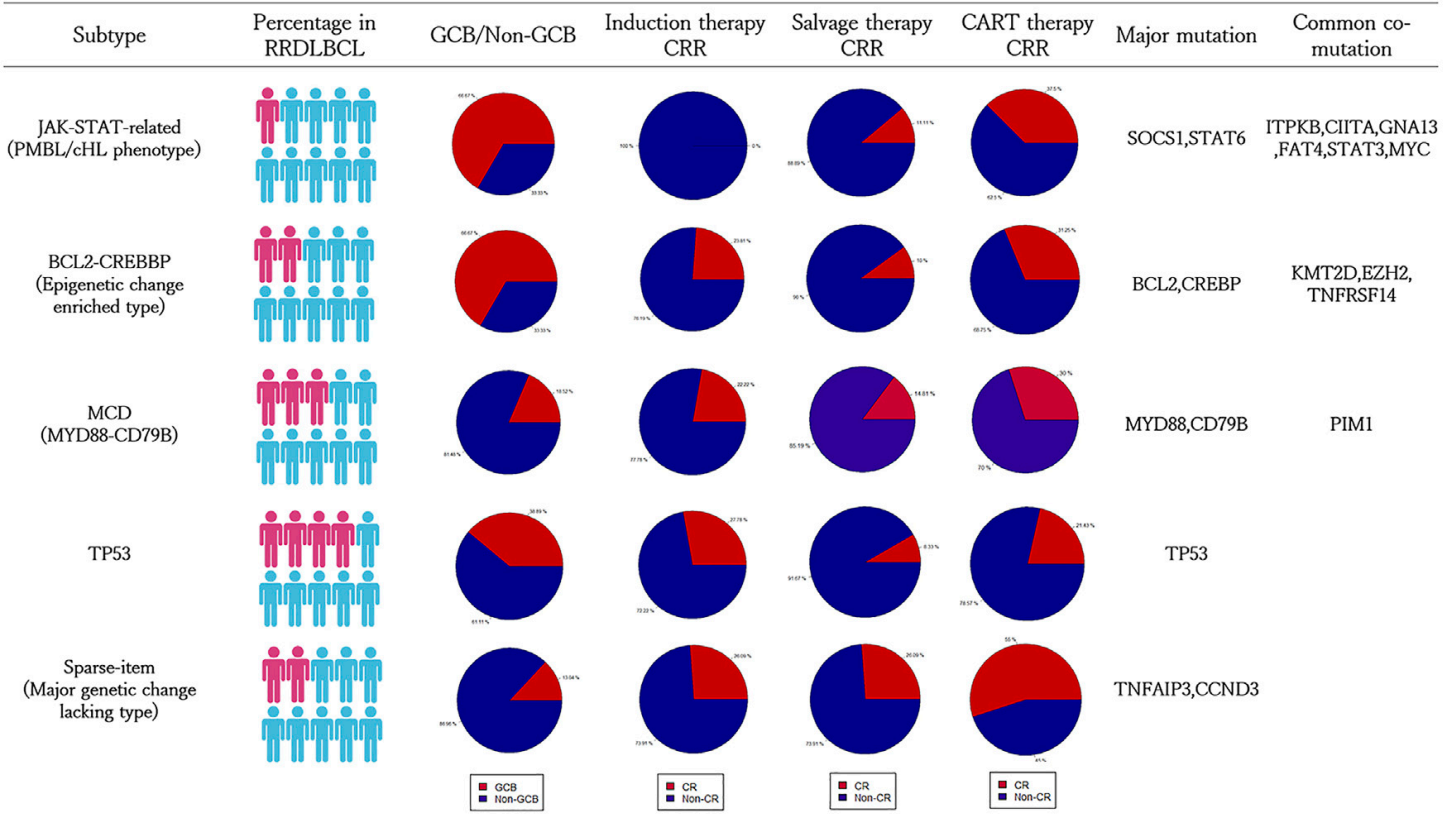
Summary of clinical information of the five subtype classification systems of RRDLBCL. The clinical information is based on 96 patients in the targeted sequencing cohort. Specific data can refer to the Supplementary Tables S2, S3.

We compared the remission rates of induction therapy, salvage therapy and CART therapy in each group. In terms of induction therapy, the CR rate of the JAK-STAT-related group was 0.0%, that is, all patients in this group responded poorly to the R-CHOP regimen, but the this difference is not statistically significant. If more common phenotypes of JAK-STAT-related types are considered (SOCS1, STAT6, ITPKB, GNA13 and CIITA), this difference is more obvious (CRR 0%, 0/12, *p* < 0.05). The salvage treatment effects of several groups were similar, with a CR rate of 10.0–20.0%. Among the patients receiving CART treatment, there were 8 cases of JAK-STAT related type, 16 cases of BCL-CREBBP, 20 cases of MCD type, 28 cases of TP53 type, and 20 cases of Sparse-item group (CART treatment refers to CD19-CAR-T or CD20-CAR-T). The proportion of each group receiving CART treatment was similar. Among them, the TP53 group had poor CART curative effect, with a remission rate of only 21.4% (6/28, *p* < 0.05); the Sparse-item group had a CART treatment remission rate of 55.0% (11/20, *p* = 0.1, Supplementary Table S3), and the curative effect was better (Figure 5). The survival analysis of each subgroup showed that the survival conditions of the MCD group (*p* < 0.1) and the TP53 group (*p* < 0.05) were significantly different than those of other patients, while the JAK-STAT-related type and BCL-CREBBP type did not show significant overall survival difference (Supplementary Figure S6). Supplementary Table S3 showed clinical information of subgroups.

### 3.5 Survival Analysis Based on Expression of High-Frequency Genes

**FIGURE 6:**
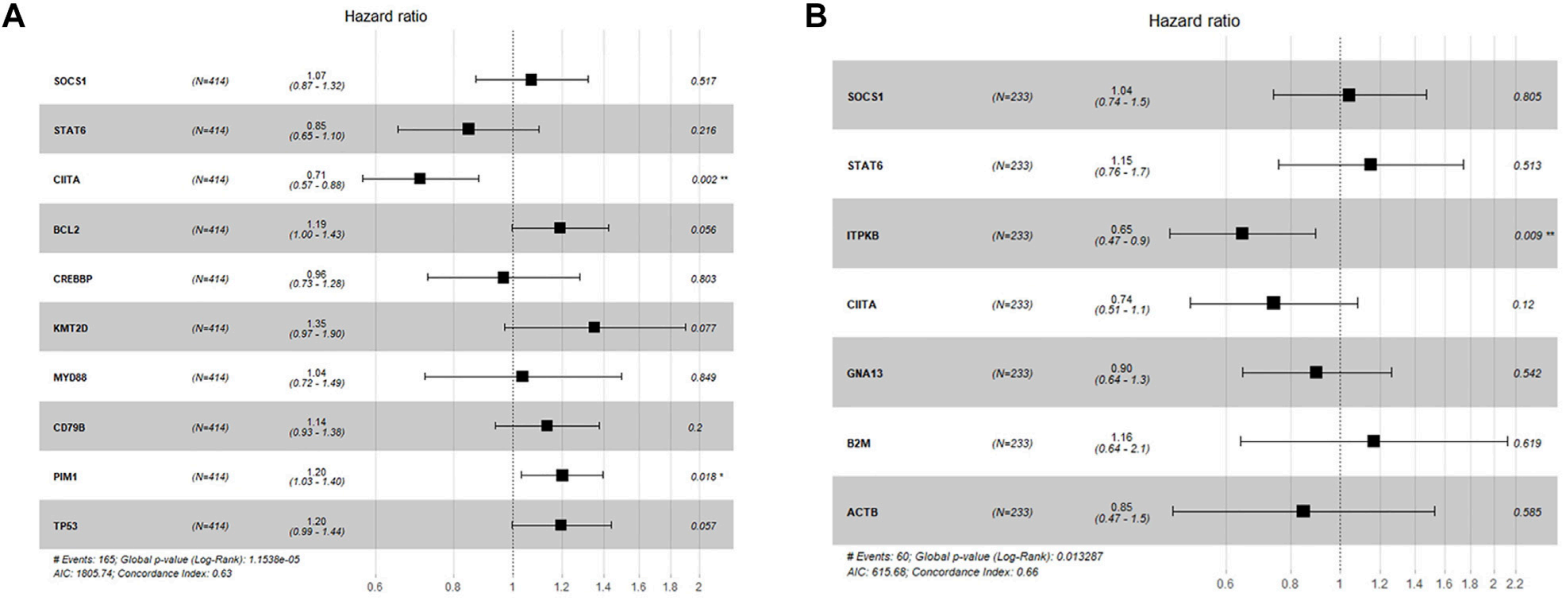
Forest plot for COX regression analysis. **(A)** Multivariate survival analysis with SOCS1, STAT6, CIITA, BCL2, KMT2D, CREBBP, PIM1, MYD88, CD79B and TP53 in the GSE10846 whole cohort. **(B)** Multivariate survival analysis with SOCS1, STAT6, CIITA, GNA13, ITPKB, B2M and ACTB in the GSE10846 R-CHOP cohort.

We used data from the same expression array to conduct survival analysis to evaluate the research meaning of the above genes. Patients were grouped according to expression levels of such genes (mean value as cut-off value, differences between high and low expression groups were significant, *p* < 0.05). Results showed that for the representative genes of each subtype, expression levels of STAT6 (*p* < 0.01), CIITA (*p* < 0.01), BCL2 (*p* < 0.05), PIM1 (*p* < 0.01) and TP53 (*p* < 0.01) had significant prognostic value in the whole GSE10846 cohort (Supplementary Figures S7A–D, *n* = 414). In the multivariate analysis performed with COX regression, PIM1 (*p* < 0.05) and CIITA (*p* < 0.01) showed most significant prognostic value (Figure 6A), and the model showed a good prediction effect (*p* < 0.001). Since it was observed that the typical JAK-STAT phenotype did not respond well to R-CHOP treatment, we further analyzed the survival significance of related genes in the R-CHOP treatment subgroup of this cohort. The results revealed that in R-CHOP treatment cohort (*n* = 233), CIITA(*p* < 0.05), ITPKB(*p* < 0.01) and GNA13 (*p* < 0.01) showed significant prognostic value (Supplementary Figures S8A–C). In the multivariate analysis performed with COX regression, ITPKB showed most significant prognostic value (*p* < 0.01), and global *p*-value of the model was 0.01 (Figure 6B).

## 4 Discussion

### 4.1 MYD88, CD79B, and PIM1: Solitary Independent MCD Subtype

MYD88 mutations and CD79A or CD79B mutations co-occur in ∼10% of ABC DLBCLs. CD79A or CD79B mutations are more prevalent in ABC DLBCLs harboring MYD88 (L265P) (34%) than in those without (18%) (Ngo et al., 2011). This indicates that these mutations have a synergistic effect in the development of ABC DLBCL. In a clinical trial, the BTK inhibitor ibrutinib created a response in 37% of ABC cases. The most significant response rate (80%) was observed in tumors with CD79B and MYD88 (L265P) mutations (Wilson et al., 2015). Phelan discovered a My-T-BCR super complex: compared with other ABC DLBCL and GCB DLBCL cell lines, the survival rate of MYD88 L265P and CD79A mutant or CD79B mutant ABC DLBCL cell lines is more dependent on this complex (Phelan et al., 2018). This mechanism can explain the connection between MYD88 and CD79B. Patients with MYD88 mutations account for a large proportion of RRDLBCL. The MCD-DLBCL proposed by Schmitz is named after MYD88 and CD79B. The results of this study also show that PIM1 is an important mutant gene in MCD DLBCL. Our research also found that among the high-frequency genes of RRDLBCL, PIM1 is most closely related to MYD88.

PIM1 was found to be an important effector of the signal transducer and activator of transcription (STAT) three and five transcription factors (Yip-Schneider et al., 1995). All PIM kinases can act as survival factors, preventing cells from undergoing apoptosis by activating BCL2 and inactivating the pro-apoptotic proteins BAD and ASK1 (Lilly et al., 1999; Aho et al., 2004; Gu et al., 2009). In DLBCL, PIM1 is a target for aberrant hypermutation, particularly in extra-nodal cases (Deutsch et al., 2007). A 2012 study by Brault showed that PIM expression in DLBCL is related to the activation of the JAK/STAT signaling pathway and proliferative activity. Correlation of nuclear PIM1 expression with disease stage, as well as a modest response to small-molecule inhibitors, suggests that PIM kinases are progression markers rather than primary therapeutic targets in DLBCL (Brault et al., 2012). In a 2018 study, PIM1 and MYD88 immunohistochemistry was performed on samples from 57 patients with PCNSL. The results suggest that PIM1 is mainly expressed in the nucleus, while MYD88 staining is largely cytoplasmic, with almost no nuclear staining. Compared with the control group, the positive expression rates of PIM1 and MYD88 were higher in PCNSL, and their expression levels were positively correlated (*r* = 0.581, *p* = 2.0 × 10−6) (Zhou et al., 2018). Whether carrying PIM1 mutations can be regarded as the MCD category and adopting similar treatment strategies as MYD88/CD79B mutations is an important question, because studies have shown that PIM1 point mutations reduce the sensitivity of ABC-DLBCL patients to ibrutinib (Kuo et al., 2016). In our research of public dataset, PIM1 and MYD88 are indeed the most closely related (6/14, 42.8%), but PIM1 does not always appear simultaneously with MYD88. At the expression level, PIM1 also showed significant correlation with CREBBP, TP53, STAT6, and KMT2D (*p* < 0.01). In the targeted sequencing cohort, the correlation between PIM1 and MYD88/CD79B appears to be weak. These results indicate that the impact of PIM1 is actually broader. Therefore, we believe that PIM1 is indeed the most common co-mutated gene of MYD88-CD79B RRDLBCL, but it is not recommended to be used as a basis for classification.

The correlation analysis in this study suggests that PRDM15 is related to PIM1 and CD79B. PRDM15 encodes a novel DNA-binding protein that regulates expression of key activators and repressors of the WNT and MAPK–ERK pathways at the chromatin level to safeguard naive pluripotency (Mzoughi et al., 2017). A recent study showed that PRDM15 regulates the transcription of key effectors of NOTCH and WNT/PCP pathways to preserve early midline structures in the developing embryo (Mzoughi et al., 2020). However, in most previous studies, PRDM1 is closely related to MCD rather than PRDM15. In our targeted sequencing association analysis, PRDM1 also showed a strong association with MYD88 (lift = 2.13).

Nevertheless, the MCD subtypes in RRDLBCL are notable, and undoubtedly a subtype with poor prognosis in DLBCL. Because the classification significance of genes such as PIM1 and PRDM1 is not conclusive, we only used MYD88/CD79B mutations as the basis for analyzing the RRDLBCL of this group in the survival analysis. Most patients in this group are Non-GCB, which is consistent with previous studies (Chapuy et al., 2018; Schmitz et al., 2018). They seem to have a worse prognosis, even when compared with patients with RRDLBCL. Of course, this difference in survival may be biased, since more patients in this group are older than 60 years (13/27, 48.1%).

### 4.2 BCL2, CREBBP and KMT2D: The Only Protagonist of EZB

In a study published in 2019, a class of molecular high-grade diffuse large B-cell lymphoma (MHG) was defined, which had significantly higher mutation frequencies than GCB in KMT2D, BCL2, MYC, or DDX3X. The progression-free survival rate at 36 months after R-CHOP in the MHG group was 37% (95% CI, 24–55%) compared with 72% (95% CI, 68–77%) for others. A 2019 NGS study on DHL and THL showed that the most frequently mutated genes were CREBBP, followed by BCL2, KMT2D, MYC and EZH2 (Evrard et al., 2019). These studies indicate that mutations in KMT2D and CREBBP appear more in the so-called high-grade DLBCL. KMT2D and CREBBP are both epigenetically regulated genes. KMT2D is a histone monomethyltransferase, which induces both monomethylated and dimethylated H3K4 (H3K4me1 and H3K4me2, respectively), thereby promoting transcription. KMT2D is mutated in about 30% of DLBCL and is one of the most frequent mutations in DLBCL (most are frameshift or nonsense mutations) (Morin et al., 2011; Schmitz et al., 2018). Integrative genomic analyses indicate that KMT2D affects methylation of lysine 4 on histone H3 (H3K4) and expression of a set of genes, including those in the CD40, JAK-STAT, Toll-like receptor, and B-cell receptor signaling pathways. At the same time, KMT2D may promote malignant outgrowth by perturbing the expression of tumor suppressor genes that control B-cell-activating pathways (Ortega-Molina et al., 2015). CREBBP is a histone acetyltransferase that mediates H3K27 acetylation and is important for gene enhancer activation. Evidence suggests that CREBBP mutations are an early event in lymphoma because mutations in this gene are also found in hematopoietic stem cells (Horton et al., 2017). CREBBP-mutant lymphomas have decreased expression of genes involved in germinal center exit, those responsible for plasma cell differentiation, and those associated with antigen presentation by MHC class II, suggesting that CREBBP deficiencies contribute to lymphomagenesis by blocking B-cell differentiation and facilitating immune escape (Jiang et al., 2017). In our study, we found that KMT2D and CREBBP mutations often appear at the same time, whether in public data or in a targeted sequencing cohort of 96 RRDLBCL patients. But there is no expression correlation between the two. BCL2 abnormal activation is usually driven by genetic abnormalities in BCL2 itself, the most important of which is the t (14; 18) (q32; q21) translocation (Miao et al., 2019). The expression level of BCL2 is sufficient to indicate the prognosis, and we have repeated this result in the study. BCL2, KMT2D, and CREBBP are the main mutations in EZB-DLBCL in the system described by Schmitz. These three genes affect about 40% of RRDLBCL patients (37/92, 40.2%) in our cohort. The EZB-DLBCL category also includes mutations such as SOCS1 and STAT6. However, we believe that in RRDLBCL, the simultaneous occurrence of SOCS1 and STAT6 with BCL2, KMT2D, and CREBBP is still relatively rare. These two sets of mutations are a manifestation of DLBCL heterogeneity, and it is necessary to distinguish them. We discuss further below.

### 4.3 STAT6 and SOCS1: Trunk of the JAK-STAT Related Subtype

In general, activation of the JAK2-STAT signaling pathway appears more in HL and PMBL (Rosenwald et al., 2003; Hartmann et al., 2008; Scott and Gandhi, 2015). HL is frequently associated with a 9p24.1 genomic amplification that includes the JAK2 locus, as well as with a cytokine-enriched tumor microenvironment. Thus, activation of the JAK2-STAT signaling pathway may promote tumor growth in HL (Navarro et al., 2009; Aldinucci et al., 2010). As for PMBL, it shares molecular features with HL (Kim et al., 2019). However, Schmitz et al. (2018) states that activators of transcription (JAK-STAT) signaling may have been promoted in 49% of cases by a STAT6 mutation or amplification or by a mutation or deletion targeting SOCS1. Lacy et al. (2020) proposed a classification system of DLBCL including SOCS1/SGK1, which is a group of DLBCL patients with mutations similar to PMBC, and the possibility of misdiagnosing PMBCL as DLBCL had been excluded through pathological review. We also believe that JAK-STAT related types should be separated in the classification of RRDLBCL, in which the most important mutations are SOCS1 and STAT6. These two mutations are strongly related, whether in the priori algorithm or at the level of expression.

In DLBCL, the SOCS1 mutation has previously been shown to be associated with good survival (Mottok et al., 2009; Schif et al., 2013). A 2016 study also showed that the SOCS1 mutations were exclusive to non-recurrent primary DLBCL and were completely absent in cases of relapsing DLBCL, which also supported a good prognosis (Juskevicius et al., 2016). However, our research shows that SOCS1 mutation still occurs in a considerable proportion of RRDLBCL, affecting about 8–20% of patients, which should not be ignored. STAT6 belongs to the STAT family of both adaptor proteins and transcription factors. STAT family members display a shared protein structure that is instrumental for their activation and functions (Miloudi et al., 2018). This family plays a key role in the proliferation and survival of B lymphocytes and is often dysregulated in lymphomas (Schwindt et al., 2009). In particular, expression and/or activation of STAT6 as well as amplification of the locus encoding STAT6 on chromosome 12 have been detected in more than 50% of PCNSL specimens (Ritz et al., 2009). In addition to PCNSL, similar to SOCS1, STAT6 mutations are also found in PMBL. An earlier study suggested that 20 of 55 (36%) PMBL cases harbor heterozygous missense mutations in the STAT6 DNA-binding domain, whereas no mutation in the gene was found in 25 diffuse large B-cell lymphoma samples (Yang et al., 2009). Some studies have shown that STAT6 is associated with poor prognosis, but the sample size is small in these cases (Mondello et al., 2020). In our clinical analysis of the targeted sequencing cohort, the overall prognosis of the JAK-STAT-related type was at a general level in RRDLBCL. However, its induction therapy had poor efficacy. Survival analysis based on expression level also suggests that typical phenotypes of JAK-STAT-related type such as CIITA, GNA13, ITPKB have significant survival significance in DLBCL, which were PMBL and cHL phenotypes as well (Steidl et al., 2011; Lees et al., 2019). The bad response of JAK-STAT-related type to RCHOP regimen suggested two possibilities: 1) JAK-STAT-related prognosis is generally good, but some patients are resistant to induction therapy, so they appear in our RRDLBCL cohort; 2)Some patients carry other mutations that may lead to poor prognosis, such as MYC, which means there is bias.

As mentioned above, some genes are closely related to STAT6 and/or SOCS1, such as ITPKB, CIITA and GNA13. In the public data cohort, because the JAK-STAT related group has more samples, it shows more meaningful association rules. ITPKB encodes for a kinase that converts the second messenger inositol trisphosphate (IP3) to IP4, a soluble antagonist of the AKT-activating PI3K-product IP3, which is the mutation most closely related to SOCS1/STAT6 (Westernberg et al., 2016). A 2019 study demonstrated that IP4 plays a critical role in redox homeostasis upon cisplatin exposure by reducing cisplatin-induced ROS through inhibition of a ROS-generating enzyme, NADPH oxidase 4 (NOX4), which promotes cisplatin-resistant tumor growth (Pan et al., 2019). Itpkb pathway inhibition increases intracellular Ca^2+^, induces apoptosis of activated T cells, and can control T-cell-mediated autoimmunity (Thangavelu et al., 2020). These mechanisms show that ITPKB is indeed worth studying in DLBCL. In our targeted sequencing cohort, ITPKB has no meaningful association rules due to its low frequency. However, in the analysis of public data, the correlation between ITPKB and SOCS1/STAT6 is very meaningful, so we still included it in the JAK-STAT related classification rules. The GNA13 mutation affected 13% (12/92) of patients in cohort of public data and showed a clear prognostic significance in the survival analysis of expression data in this study. Previous studies suggest that GNA13, which is related to cell migration (Green et al., 2011), is the most common mutant gene in germinal center (GC)-derived B-cell lymphoma, including nearly a quarter of Burkitt’s lymphoma and GC-derived diffuse large B-cell lymphoma cases (Zhang et al., 2013). And GNA13 loss is associated with GC B-cell persistence, which may lead to an increased risk of lymphoma development (Healy et al., 2016). Xia et al. (2021) demonstrated that inactivating GNA13 by targeting its palmitoylation enhanced the sensitivity of GCB-DLBCL to the BCL2 inhibitor, which further showed the clinical significance of the gene. Research into the role of CIITA in lymphoma has focused on gray zone lymphoma (GZL) (Eberle et al., 2011). A 2019 study revealed that cases of GZL with LBCL-like morphology had more PD-L1/PD-L2 or CIITA rearrangements than cHL-like cases, which presented genetic immune escape features (Sarkozy et al., 2019). In addition, the analysis of the targeted sequencing cohort also showed that STAT3 and CD58 are common gene mutations of JAK-STAT related type as well, but the frequency of these mutations is lower.

### 4.4 RRDLBCL Classification and Precision Medicine

Our research relies on part of the published research data, and there is an inevitable lack of information. For example, the NOTCH2 mutation was discarded in the process of dimensionality reduction because of a significant false positive in one of the data sources. However, we repeated the classification method with a targeted sequencing cohort and verified the clinical significance of the system. Overall, the results of this study are beneficial for the classification and treatment of RRDLBCL. In terms of classification, the so-called MCD and EZB types cover about 2/3 of RRDLBCL. In EZB classification, we believe that it is meaningful to distinguish mutations related to the JAK-STAT pathway from mutations represented by BCL2 and CREBBP; this refines classification of RRDLBCL and provides guidance for further research. In sum, RRDLBCL is classified into five types, the clinical characteristics of each subtype can be seen in Figure 5, and the pathways and mechanisms involved are shown in Supplementary Figure S9:

1) JAK-STAT related type: including STAT6 and SOCS1. Common co-mutations of this subtype include CIITA, ITPKB, FAT4, STAT3, MYC and B2M. The mutation lineage is similar to PMBL and cHL. This type involves many mechanisms, such as cell migration and immune escape. However, in general, there is a close relationship with the JAK-STAT pathway or the interferon-gamma-mediated signaling pathway, which was revealed by GO analysis and is a upstream pathway of JAK-STAT. The clinical evidence we provide shows that the main reason for this subtype to be relapsed and refractory is the failure of induction therapy.
2) BCL-CREBBP type (Epigenetic change enriched type): The main characteristics are genetic changes in BCL2 and enriched epigenetic mutations. Epigenetic mutations such as CREBBP and KMT2D are more common in this type, and are often accompanied by BCL2. Other common co-mutations of this subtype include TNFRSF14 and EZH2.
3) MCD type: MYD88 and CD79B mutations are the main genetic changes of this type. These genes are involved in the BCR signaling and related pathways, and are connected with the common NF-κB pathway. This is the most independent category, with a more conservative mutant gene lineage. PIM1 mutations are more common in this subtype.
4) TP53 mutation: Patients with TP53 mutant. TP53 is a relatively independent mutation and lacks other mutations associated with it. The TP53 mutation suggests a worse prognosis even in the RRDLBCL population and a poor response to CART treatment.
5) Undefined type (Sparse-item type/Major genetic change lacking type): Refers to a subtype of patients who lack the above-mentioned major genetic changes and cannot be classified into the above-mentioned categories. This part of patients has no obvious genetic characteristics. Although the frequency of TNFAIP3 and CCND3 mutations was found to be higher in the targeted sequencing cohort, this feature was not observed in the public data set. Despite the lack of genetic markers, since this subtype represents the group of patients with the best overall survival and is an excellent candidate for CART therapy, it is still valuable to classify it.

Compared with the previous large-scale genetic research, the classification method of this study mainly recommends distinguishing SOCS1/STAT6 from EZB, because it is related to a series of PMBL/cHL phenotypes, which show poor efficacy on RCHOP. In the studies of Lacy et al. (2020) and Wright et al. (2020), the significance of TET2/SGK1 as a classification factor was mentioned, but in the two cohorts of this study, these two mutations are completely independent (TET2/SGK1 co-mutant only occurred in one patient in both cohorts), which suggests that the mutation lineages of RRDLBCL and *de novo* DLBCL are still different. The distribution patterns of Chinese and western patients are basically similar. In the Chinese patient cohort of this study, the mutation rate of TP53 is higher and there is more overlap between TP53 and MYD88, but this may be due to the bias of the inclusion of patients, for patients who tend to agree to NGS in China usually experience a more tortuous treatment process and their genetic changes could be worse or more complicated. Due to the low mutation frequency of NOTCH1 and NOTCH2, it is difficult to effectively analyze the role of NOTCH pathway in RRDLBCL in this study. In the targeted sequencing cohort, there were only four cases (4/96, 4.2%) of NOTCH1 and five cases (5/96, 5.2%) of NOTCH2, and one case carried both. Since previous studies have proved the significance of genetic changes in the NOTCH pathway in DLBCL (Rossi et al., 2011; Chigrinova et al., 2013; Fabbri et al., 2013; Schmitz et al., 2018), we believe that this is one of the shortcomings of this study, but the aforementioned five-subtype classification should be sufficient to provide better guidance for the diagnosis and treatment of RRDLBCL.

Currently, salvage immunochemotherapy and autologous hematopoietic stem cell transplantation (ASCT) are still the preferred treatment options for RRDLBCL (NCCN, 2018). However, there are patients who are not suited to receive ASCT, and who find it difficult to achieve remission by general immunochemotherapy. In this context, many optional chimeric antigen receptor T-cell (CAR-T) immunotherapies, other immunotherapies, and targeted therapies make individualized treatment options complicated and subjective. By further classifying RRDLBCL based on mutations, combined with the pathological classification of GCB and ABC, the choice of treatment may become more directional and individualized. As mentioned, the ibrutinib response rate is comparatively good for MCD DLBCL (Ngo et al., 2011). JAK-STAT related types share similar gene mutation profiles with cHL, and there is already evidence that patients with relapsed and refractory cHL can benefit from treatment of anti-human PD-1 monoclonal antibodies (Younes et al., 2016; Chen et al., 2019). Therefore, we anticipate that immune checkpoint inhibitors may also improve the prognosis of these DLBCL patients. In addition, JAK-STAT related subtype may also become candidates for CART therapy. TP53 mutation is of great significance in the classification and evaluation of RRDLBCL. Recent research indicated that INCB057643 combining with the BCL-2 inhibitor venetoclax displayed potent therapeutic synergy in DLBCL/HGBCL cells with and without concurrent TP53 mutation, which may bring new treatment options to patients with TP53 mutation (Deng et al., 2021). Moreover, due to the low complete remission rate of CART treatment, clinical trials and allogeneic hematopoietic stem cell transplantation may be considered more positively for patients with TP53 mutant. On the other hand, our research suggests that CART therapy may be a better choice for patients who lack major genetic changes (The Sparse-item subtype).

## 5 Conclusion

As a summary, with analysis of 92 RRDLBCL public WES data and 96 Chinese RRDLBCL patients’ targeted sequencing data, we believe that RRDLBCL can be divided into five main types: 1) JAK2-STAT-related type: including STAT6, SOCS1, ITPKB, CIITA, etc. The mutation lineage is similar to PMBL and cHL. 2) EZB type: Epigenetic mutations such as KMT2D and CREBBP are more common in this type, and are often accompanied by BCL2 and EZH2 mutations. 3) MCD type: including MYD88 and CD79B, PIM1 is more common in this subtype. 4) TP53 mutation:TP53 mutant patients. 5) Undefined type (Sparse Mutation type): We prefer to call it Major Genetic Change Lacking Type, which has a better prognosis and better response to CART treatment. For JAK-STAT-related type RRDLBCL, further pathological studies and molecular biology studies are needed to evaluate its significance as an independent classification. In addition, among the common gene mutations, the expression changes in BCL2, PIM1, STAT6, TP53 have significant prognostic significance in the whole GSE10846 cohort, and PMBL/cHL phenotype such as ITPKB, CIITA and GNA13 proved to have predictive significance for the outcome of RCHOP regimen. Based on our analysis, the distribution of RRDLBCL genetic changes in the Chinese population is basically consistent with the published studies. And these analysis results may be helpful for establishing a complete genetic classification system suitable for clinical use.

## Data Availability

The original contributions presented in the study are included in the article/Supplementary Material, further inquiries can be directed to the corresponding author.
