# Supplementary material for: Genetic Landscape of Relapsed and Refractory Diffuse Large B-Cell Lymphoma: A Systemic Review and Association Analysis With Next-Generation Sequencing
Source: Front Genet. 2021 Dec 2;12:677650. doi: 10.3389/fgene.2021.677650 (PMC8675234; doi:10.3389/fgene.2021.677650)
Supplement: Supplementary file 1 [file DataSheet3.docx]

Supplementary Material 2

# Supplementary Figures and Tables

## Supplementary Figures


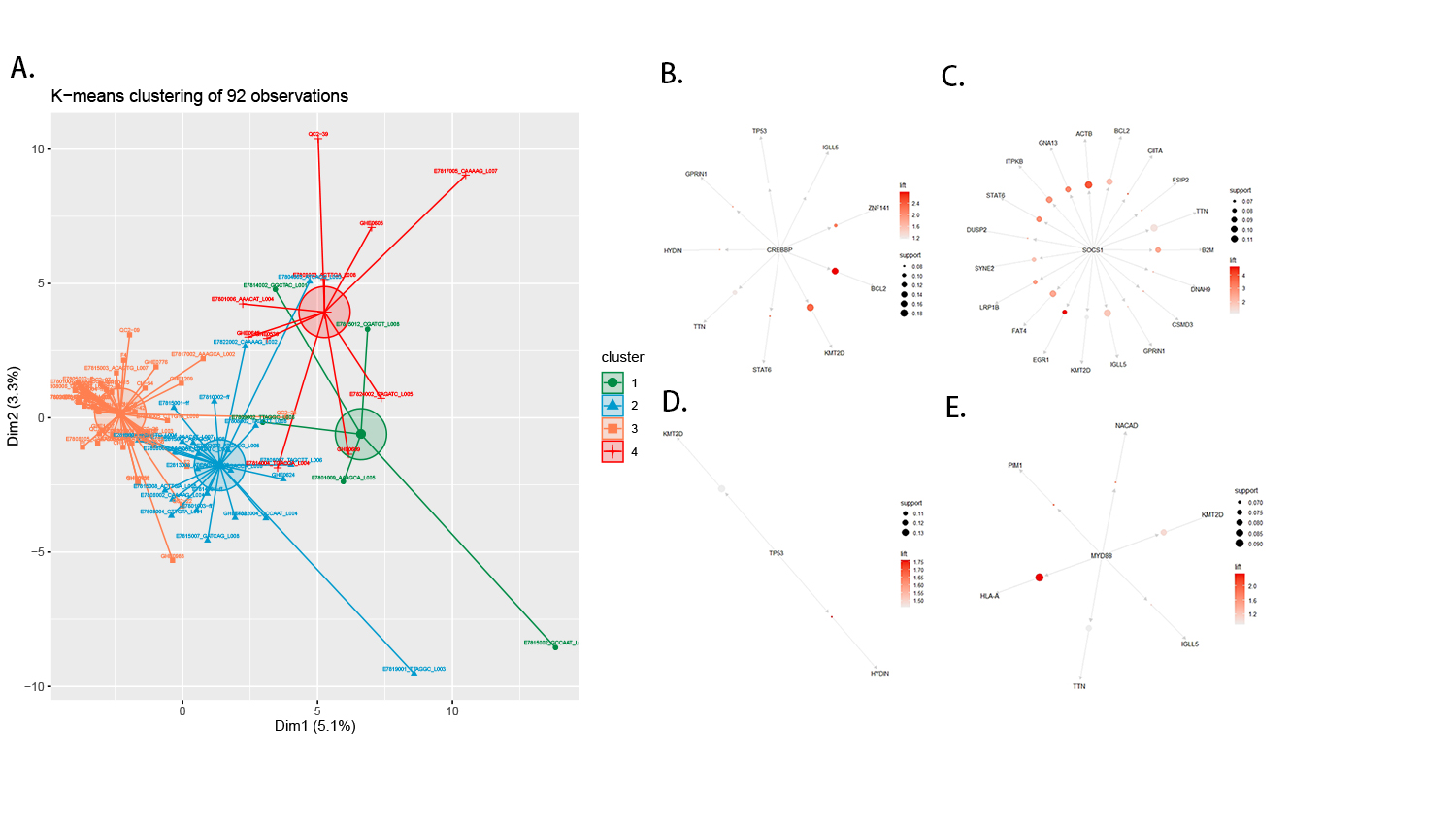


**FigureS1.** (A) Graphical representation of K-means clustering results for 92 observations with 256 genes. (B) Visualization of association rules of CREBBP. (C) Visualization of association rules of SOCS1. (D) Visualization of association rules of TP53. (E) Visualization of association rules of MYD88.


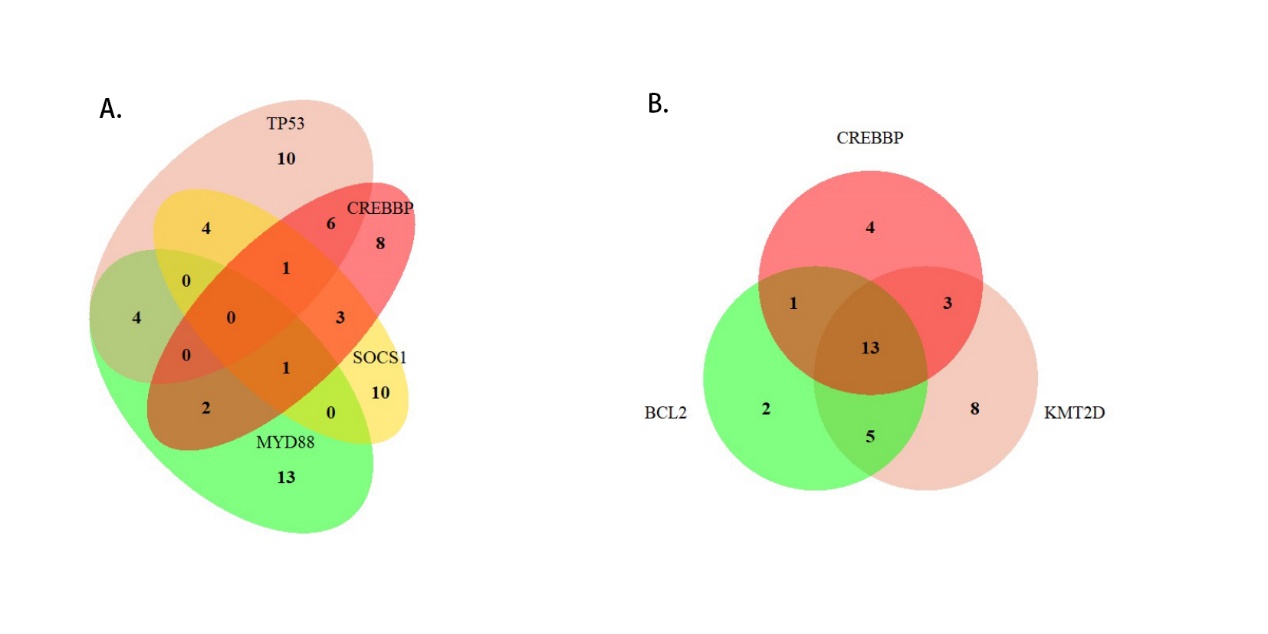


**FigureS2.** Venn diagrams of some hotspot genes: (A) Venn diagrams of SOCS1, CREBBP, TP53 and MYD88; (B) Venn diagrams of BCL2, CREBBP and KMT2D.


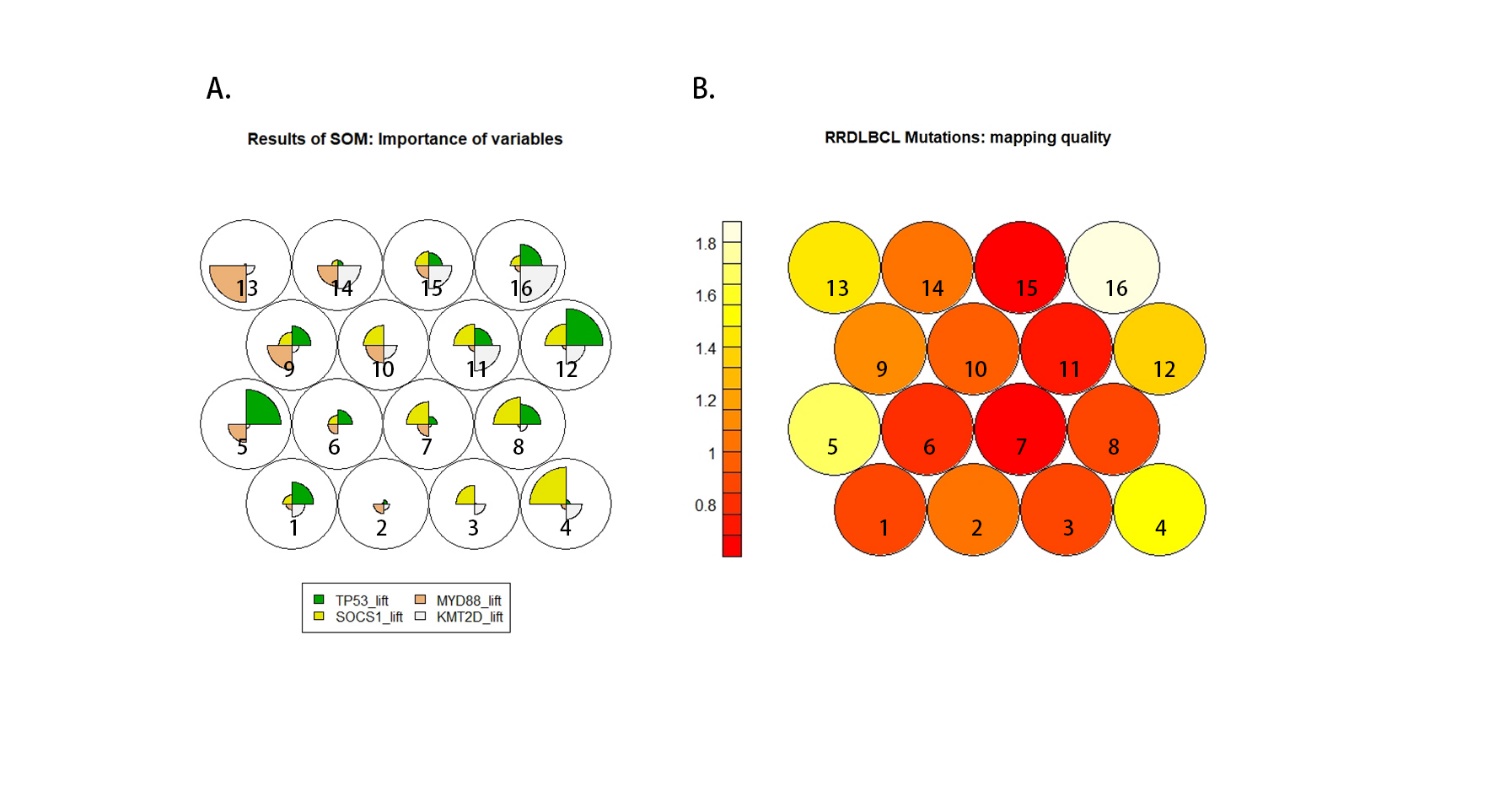


**FigureS3.** (A)Cluster analysis of SOM self-organizing network for 241 genes (Some genes with weak associations with all variable genes are discarded). Each circle represents a cluster, and the size of the sector represents the strength of the association. (B)Evaluation results of SOM neural network clustering. The clusters are numbered 1-16 from left to right and from bottom to top. The color depth represents the mean distance of various observation samples from the center. The vertical axis represents the average distance, which is used to measure the quality of clustering.


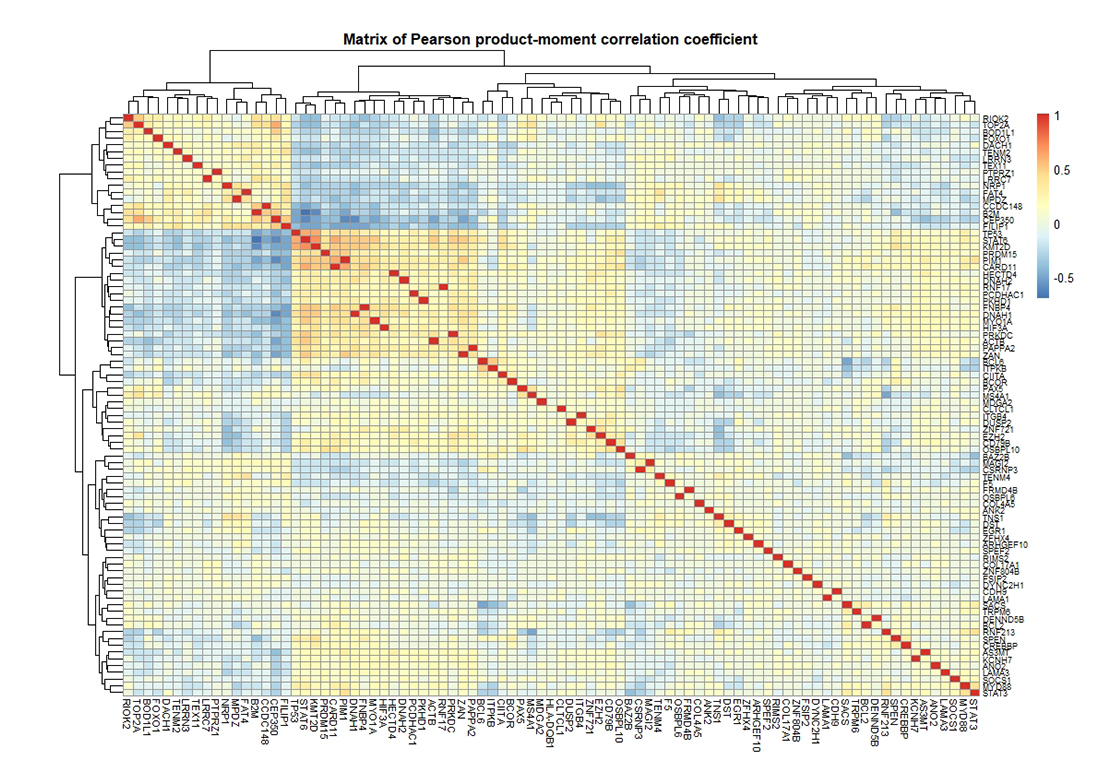


**FigureS4.** Matrix heat map of Pearson correlation coefficient among 241 genes in RNA-seq with GSE10846 cohort.


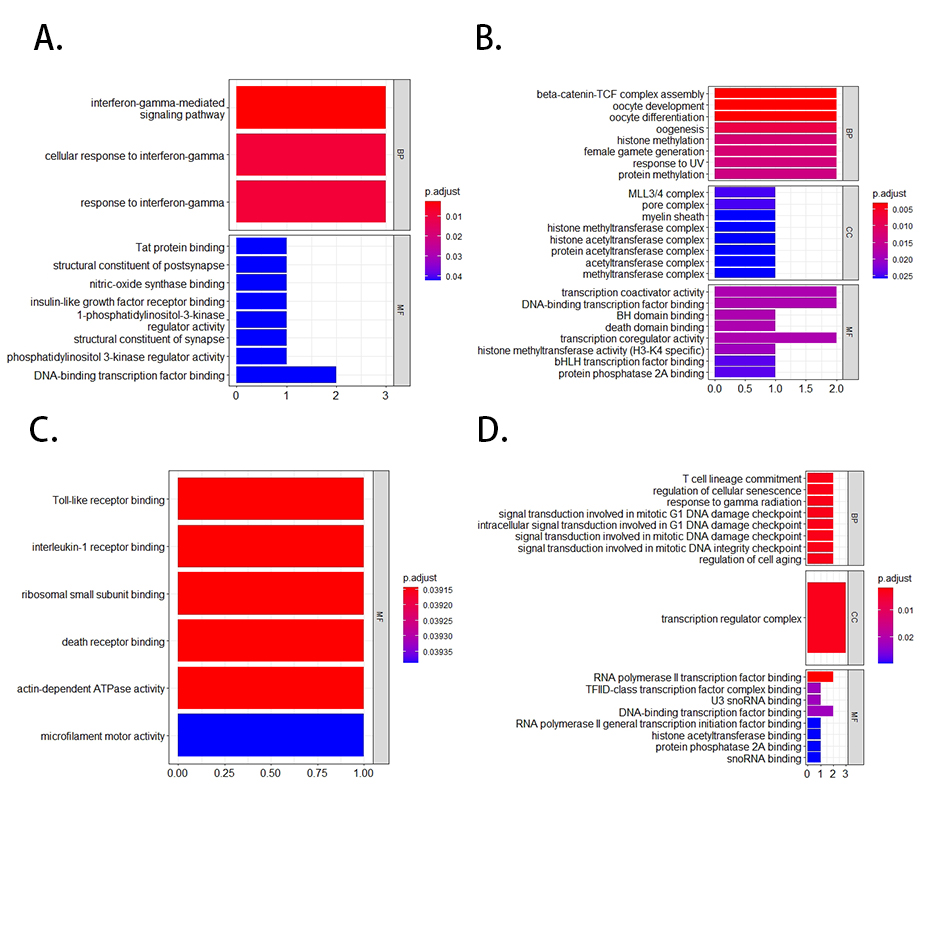


**FigureS5.** GO analysis results of Type 1-4.


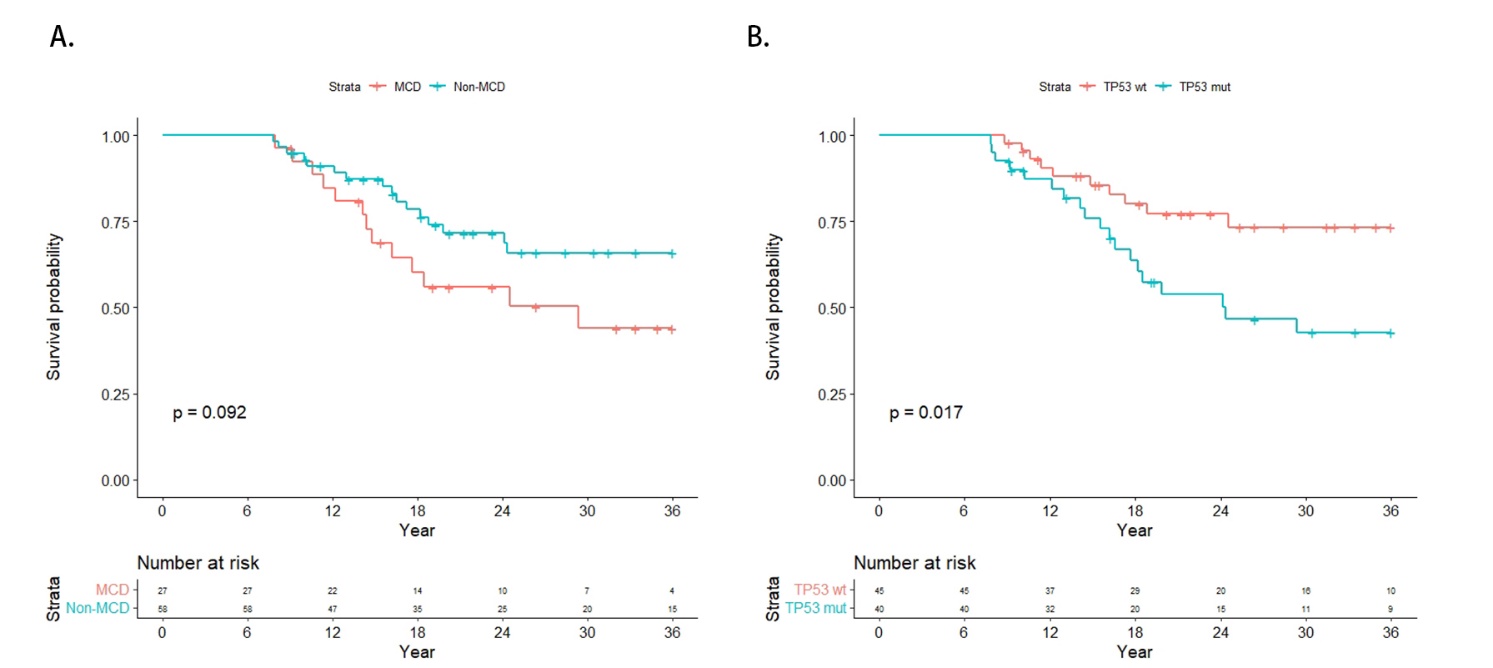


**FigureS6.** Survival analysis grouped according to four subtypes in 85 cases of RRDLBCL. (A) Survival analysis based on whether it is MCD type; (B) Survival analysis based on whether it is TP53 mutation.


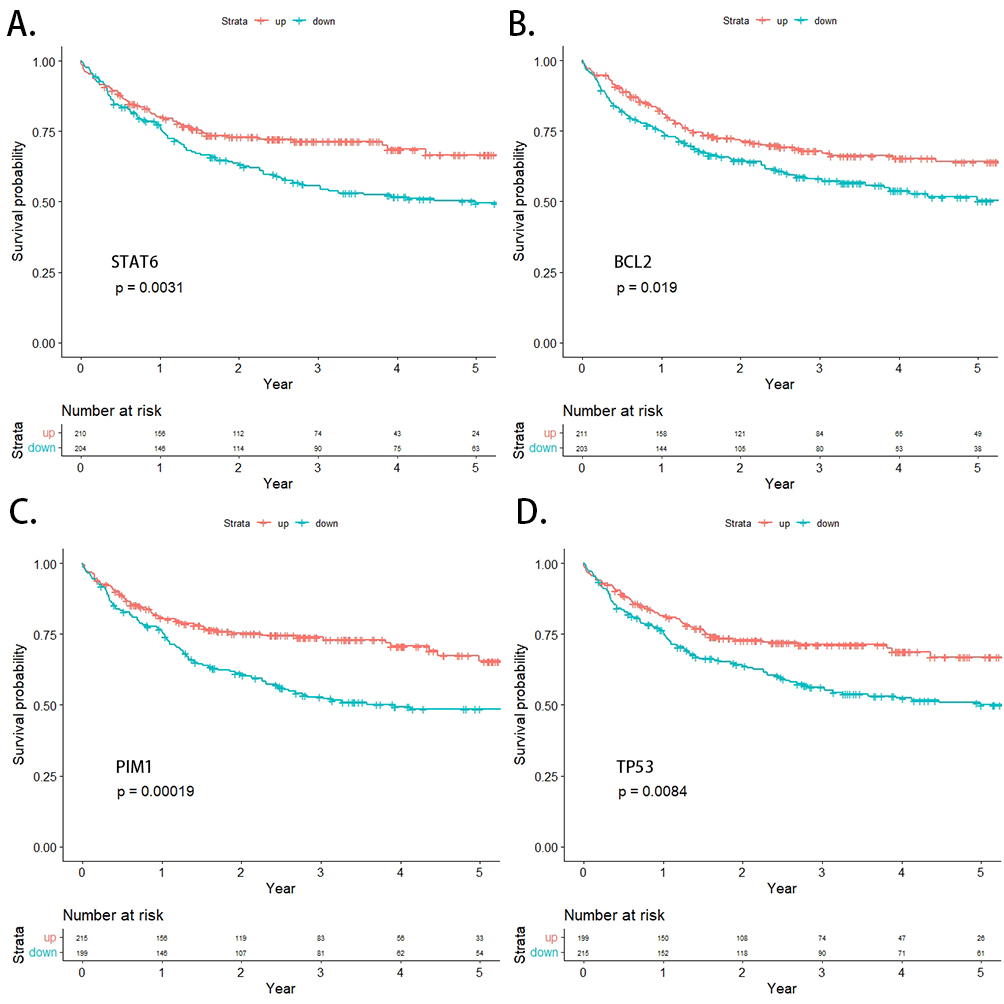


**FigureS7.** Survival analysis grouped according to several gene expression levels in whole GSE10846 cohort. (A) Survival curve according to STAT6 expression level; (B) Survival curve according to BCL2 expression level; (C) Survival curve according to PIM1 expression level; (D) Survival curve according to TP53 expression level.


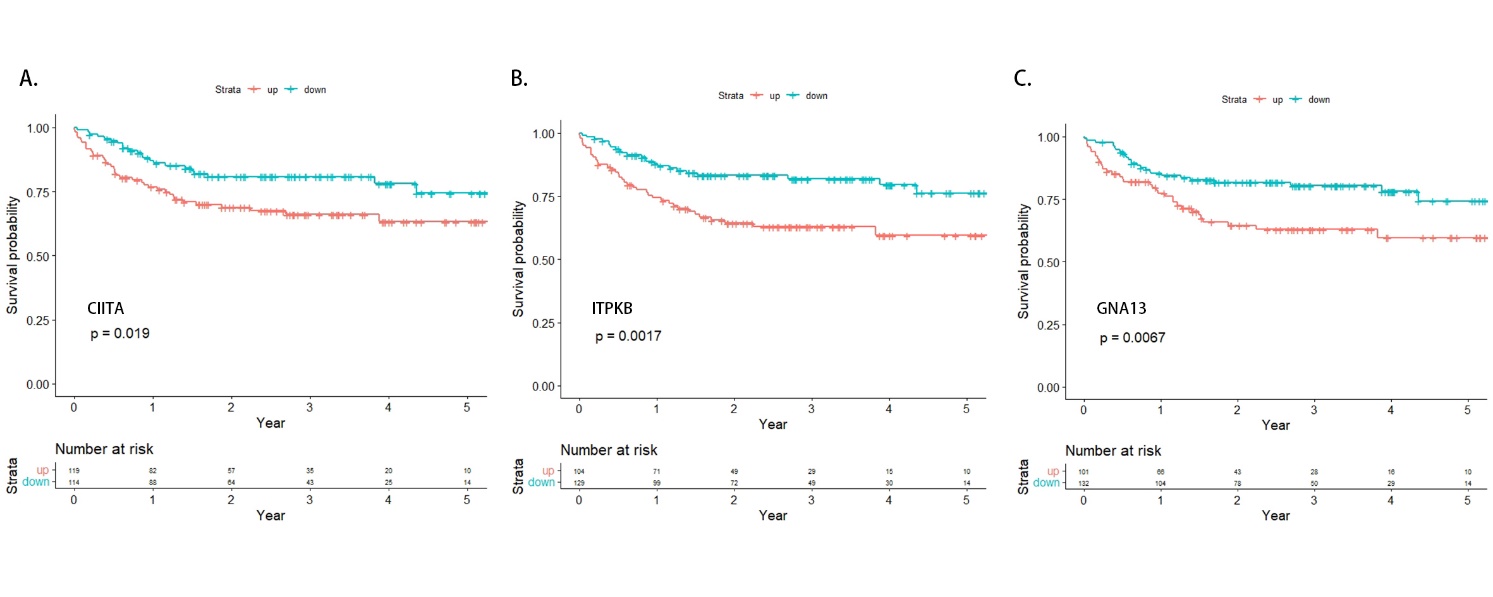


**FigureS8.** Survival analysis grouped according to several gene expression levels in GSE10846 R—CHOP cohort. (A) Survival curve according to CIITA expression level; (B) Survival curve according to ITPKB expression level; (C) Survival curve according to GNA13 expression level.


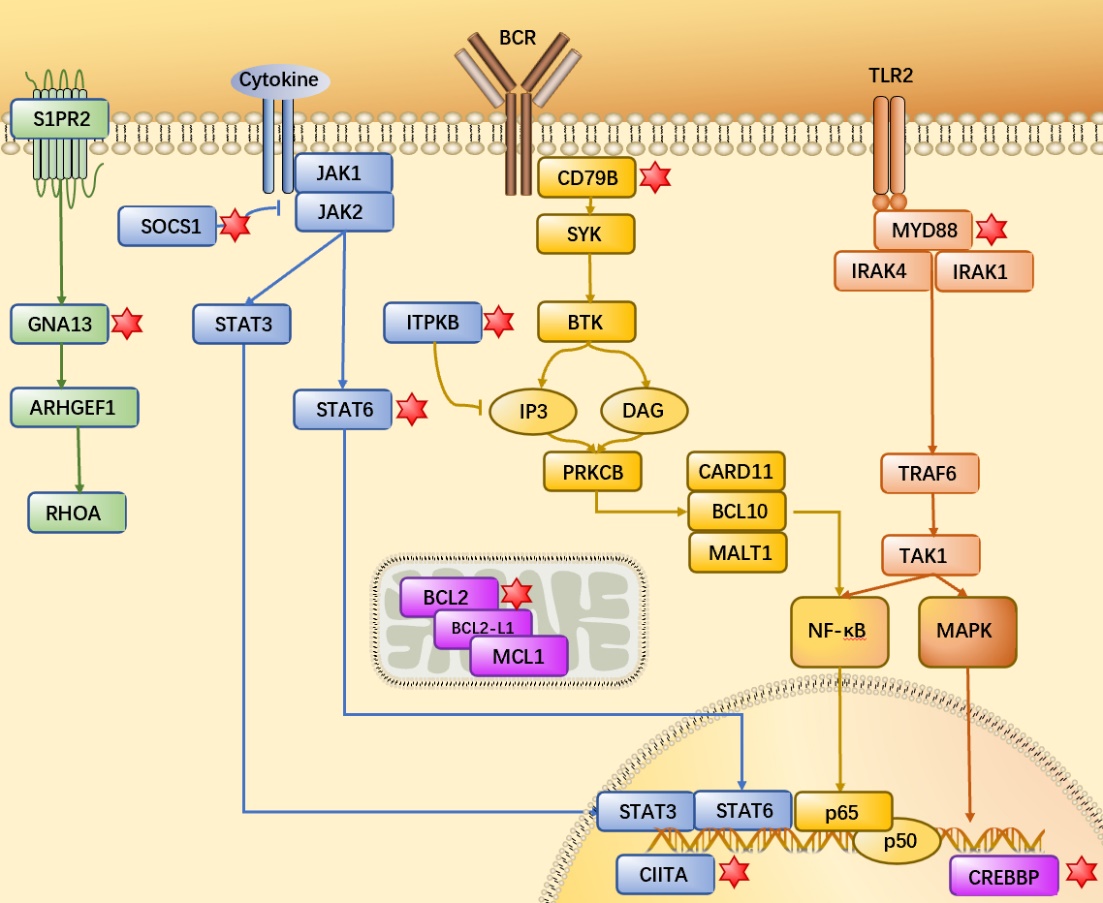


**FigureS9.** Schematic diagram of the main mutations of RRDLBCL and the pathways and mechanisms involved. Red stars mark some of the highest-frequency mutations in RRDLBCL.

## Supplementary Tables

**Table S1.** SOM self-organizing network cluster analysis results.

| **Gene** | **TP53_lift** | **SOCS1_lift** | **MYD88_lift** | **KMT2D_lift** | **cluster** | **Num*** |
| --- | --- | --- | --- | --- | --- | --- |
| HYDIN | 1.76 | 1.029239766 | 0.977777778 | 1.685823755 | 1 | 18 |
| FAT1 | 1.1 | 1.447368421 | 1.1 | 1.137931034 | 1 | 16 |
| DNHD1 | 1.408 | 1.852631579 | 0.88 | 1.213793103 | 1 | 10 |
| NEB | 1.408 | 1.389473684 | 0.44 | 0.910344828 | 1 | 10 |
| COL22A1 | 1.76 | 0.926315789 | 0.88 | 1.213793103 | 1 | 10 |
| HSPG2 | 1.408 | 0.926315789 | 0.88 | 1.213793103 | 1 | 10 |
| COL6A6 | 1.32 | 1.157894737 | 0.55 | 1.137931034 | 1 | 8 |
| MUC19 | 1.508571429 | 1.323308271 | 0.628571429 | 1.300492611 | 1 | 7 |
| IRF8 | 1.508571429 | 1.323308271 | 0 | 0.866995074 | 1 | 7 |
| DNAH10 | 1.508571429 | 0.661654135 | 0.628571429 | 1.733990148 | 1 | 7 |
| HLA-B | 1.005714286 | 0.661654135 | 0.628571429 | 1.300492611 | 1 | 7 |
| INPP4B | 1.005714286 | 0.661654135 | 0.628571429 | 1.300492611 | 1 | 7 |
| MROH2A | 1.508571429 | 0.661654135 | 0 | 1.300492611 | 1 | 7 |
| ADGB | 2.011428571 | 0 | 0 | 1.300492611 | 1 | 7 |
| GRIA2 | 1.76 | 1.543859649 | 0.733333333 | 1.011494253 | 1 | 6 |
| MYH4 | 1.173333333 | 1.543859649 | 0.733333333 | 1.011494253 | 1 | 6 |
| PTPN14 | 1.173333333 | 0.771929825 | 0.733333333 | 1.011494253 | 1 | 6 |
| PTPRF | 1.173333333 | 0.771929825 | 0.733333333 | 1.011494253 | 1 | 6 |
| TDRD6 | 1.76 | 0.771929825 | 0.733333333 | 1.011494253 | 1 | 6 |
| PREX2 | 1.76 | 0 | 0.733333333 | 1.011494253 | 1 | 6 |
| IRF2BP2 | 1.408 | 1.852631579 | 0.88 | 1.213793103 | 1 | 5 |
| CRYBG3 | 1.408 | 0.926315789 | 0 | 1.213793103 | 1 | 5 |
| AARS2 | 1.76 | 1.157894737 | 0 | 1.517241379 | 1 | 4 |
| CEP192 | 1.76 | 1.157894737 | 1.1 | 1.517241379 | 1 | 4 |
| NLRP4 | 0.88 | 0 | 0 | 1.517241379 | 1 | 4 |
| IRS4 | 0 | 0.661654135 | 1.257142857 | 1.300492611 | 2 | 7 |
| CCND3 | 0 | 0 | 1.257142857 | 0.433497537 | 2 | 7 |
| DSC2 | 0.704 | 0.926315789 | 0.88 | 1.213793103 | 2 | 5 |
| MAGEC3 | 0 | 0.926315789 | 0.88 | 0.606896552 | 2 | 5 |
| ABLIM3 | 0.704 | 0 | 0.88 | 1.213793103 | 2 | 5 |
| PTPRB | 0.704 | 0 | 0.88 | 1.213793103 | 2 | 5 |
| SLITRK5 | 0.704 | 0 | 0.88 | 1.213793103 | 2 | 5 |
| CHRNA4 | 0 | 0 | 0.88 | 0.606896552 | 2 | 5 |
| PCDHB5 | 0.88 | 0 | 1.1 | 0.75862069 | 2 | 4 |
| TDO2 | 0.88 | 0 | 0 | 0.75862069 | 2 | 4 |
| TENM3 | 0 | 0 | 1.1 | 0.75862069 | 2 | 4 |
| FAT4 | 0.621176471 | 2.452012384 | 0 | 1.427991886 | 3 | 17 |
| MAGI2 | 0.782222222 | 1.543859649 | 0.488888889 | 1.011494253 | 3 | 9 |
| BOD1L1 | 0.586666667 | 2.315789474 | 0 | 1.011494253 | 3 | 6 |
| FOXO1 | 0.586666667 | 1.543859649 | 0 | 1.011494253 | 3 | 6 |
| SACS | 0 | 1.852631579 | 0 | 1.213793103 | 3 | 5 |
| TENM2 | 0 | 1.852631579 | 0 | 1.213793103 | 3 | 5 |
| FNBP4 | 0 | 1.852631579 | 0 | 0.606896552 | 3 | 5 |
| COL17A1 | 0 | 2.315789474 | 0 | 1.517241379 | 3 | 4 |
| RIMS2 | 0 | 2.315789474 | 0 | 0.75862069 | 3 | 4 |
| DACH1 | 0 | 1.157894737 | 0 | 1.517241379 | 3 | 4 |
| ARHGEF10 | 0 | 1.157894737 | 0 | 0.75862069 | 3 | 4 |
| SOCS1 | 0.926315789 | 4.631579 | 0.231578947 | 1.117967332 | 4 | 19 |
| ITPKB | 0.234666667 | 2.778947368 | 0.586666667 | 1.213793103 | 4 | 15 |
| STAT6 | 0.541538462 | 2.850202429 | 0.338461538 | 1.400530504 | 4 | 13 |
| ACTB | 0.88 | 3.859649123 | 0.733333333 | 0.75862069 | 4 | 12 |
| EGR1 | 1.005714286 | 4.631578947 | 0 | 1.300492611 | 4 | 7 |
| PTPRZ1 | 0.586666667 | 3.859649123 | 0.733333333 | 1.011494253 | 4 | 6 |
| ZFHX4 | 0 | 3.859649123 | 0.733333333 | 1.011494253 | 4 | 6 |
| LRRN3 | 0.586666667 | 3.087719298 | 0.733333333 | 1.517241379 | 4 | 6 |
| CEP350 | 0.704 | 2.778947368 | 0.88 | 1.213793103 | 4 | 5 |
| HIF3A | 0 | 3.473684211 | 0 | 2.275862069 | 4 | 4 |
| F5 | 0 | 3.473684211 | 1.1 | 1.517241379 | 4 | 4 |
| MS4A1 | 0 | 3.473684211 | 0 | 1.517241379 | 4 | 4 |
| ASPM | 1.564444444 | 0.514619883 | 1.466666667 | 1.011494253 | 5 | 9 |
| DPP10 | 2.64 | 0 | 2.2 | 0.75862069 | 5 | 4 |
| HEATR5B | 3.52 | 0 | 1.1 | 0.75862069 | 5 | 4 |
| LNX1 | 1.76 | 0 | 2.2 | 0.75862069 | 5 | 4 |
| OR2L5 | 1.76 | 0 | 1.1 | 0.75862069 | 5 | 4 |
| TMSB4X | 1.173333333 | 0.61754386 | 1.173333333 | 0.809195402 | 6 | 15 |
| HSP90AB1 | 1.508571429 | 0.661654135 | 0.942857143 | 0.650246305 | 6 | 14 |
| FAT3 | 1.466666667 | 1.157894737 | 1.466666667 | 0.75862069 | 6 | 12 |
| CCDC40 | 0.96 | 1.684210526 | 0.8 | 0.275862069 | 6 | 11 |
| CD58 | 1.28 | 0.842105263 | 1.2 | 0.551724138 | 6 | 11 |
| RB1 | 1.056 | 1.389473684 | 0.44 | 0.606896552 | 6 | 10 |
| FAS | 0.782222222 | 1.029239766 | 0.977777778 | 1.011494253 | 6 | 9 |
| PCDH15 | 1.173333333 | 1.029239766 | 0.488888889 | 0.337164751 | 6 | 9 |
| ADAMTSL1 | 0.88 | 1.157894737 | 1.1 | 0.75862069 | 6 | 8 |
| MPEG1 | 1.173333333 | 0.771929825 | 1.466666667 | 0.505747126 | 6 | 6 |
| ANK3 | 0.704 | 0.926315789 | 0.88 | 0.606896552 | 6 | 5 |
| BAGE2 | 1.408 | 0.926315789 | 0.88 | 0.606896552 | 6 | 5 |
| RBL2 | 0.704 | 0.926315789 | 1.76 | 0.606896552 | 6 | 5 |
| ZEB2 | 0.704 | 0.926315789 | 1.76 | 0.606896552 | 6 | 5 |
| PPP2R3A | 1.408 | 0 | 0.88 | 0.606896552 | 6 | 5 |
| ATP8B2 | 0.88 | 1.157894737 | 0 | 0.75862069 | 6 | 4 |
| HVCN1 | 0.88 | 1.157894737 | 0 | 0.75862069 | 6 | 4 |
| TRANK1 | 0.88 | 1.157894737 | 0 | 0.75862069 | 6 | 4 |
| VCAN | 0.88 | 1.157894737 | 1.1 | 0.75862069 | 6 | 4 |
| IGLL5 | 0.704 | 1.852631579 | 1.056 | 1.092413793 | 7 | 25 |
| LRP1B | 0.586666667 | 2.701754386 | 1.466666667 | 0.505747126 | 7 | 12 |
| PCLO | 1.28 | 2.105263158 | 1.2 | 1.103448276 | 7 | 11 |
| ACTG1 | 1.056 | 2.315789474 | 1.76 | 0.910344828 | 7 | 10 |
| BTG2 | 0.391111111 | 2.573099415 | 0.977777778 | 0.674329502 | 7 | 9 |
| MYC | 0.782222222 | 2.058479532 | 0.977777778 | 0.674329502 | 7 | 9 |
| NRK | 0.44 | 1.736842105 | 1.1 | 0.75862069 | 7 | 8 |
| ARID1B | 0.586666667 | 2.315789474 | 0.733333333 | 1.011494253 | 7 | 6 |
| DMXL2 | 0 | 2.315789474 | 0.733333333 | 0.505747126 | 7 | 6 |
| MYBL1 | 0.586666667 | 2.315789474 | 0.733333333 | 0.505747126 | 7 | 6 |
| DCHS1 | 0.704 | 1.852631579 | 1.76 | 0.606896552 | 7 | 5 |
| DDX3X | 0.88 | 2.315789474 | 1.1 | 0.75862069 | 7 | 4 |
| IKZF3 | 0.88 | 2.315789474 | 1.1 | 0.75862069 | 7 | 4 |
| PCDH17 | 0.88 | 2.315789474 | 1.1 | 0.75862069 | 7 | 4 |
| PIM2 | 0 | 2.315789474 | 1.1 | 0.75862069 | 7 | 4 |
| TGM5 | 0.88 | 2.315789474 | 1.1 | 0.75862069 | 7 | 4 |
| B2M | 1.173333333 | 2.470175439 | 0.586666667 | 1.011494253 | 8 | 15 |
| FSIP2 | 1.28 | 2.526315789 | 0.4 | 1.103448276 | 8 | 11 |
| DNAH2 | 1.28 | 2.105263158 | 0.8 | 0.827586207 | 8 | 11 |
| CIITA | 1.32 | 3.473684211 | 0.55 | 0.75862069 | 8 | 8 |
| DNAH1 | 1.32 | 2.315789474 | 0 | 1.137931034 | 8 | 8 |
| RNF17 | 1.32 | 2.315789474 | 1.1 | 1.137931034 | 8 | 8 |
| TNS1 | 1.32 | 1.736842105 | 0 | 0.75862069 | 8 | 8 |
| PAX5 | 1.005714286 | 2.646616541 | 0 | 0.866995074 | 8 | 7 |
| RNF213 | 1.173333333 | 3.087719298 | 0 | 0.505747126 | 8 | 6 |
| BCL6 | 1.408 | 2.778947368 | 0.88 | 1.213793103 | 8 | 5 |
| FRMD4B | 1.408 | 2.778947368 | 0.88 | 1.213793103 | 8 | 5 |
| LAMA3 | 1.408 | 2.778947368 | 0 | 1.213793103 | 8 | 5 |
| ANO2 | 0.704 | 2.778947368 | 0 | 0.606896552 | 8 | 5 |
| HECTD4 | 1.408 | 2.778947368 | 0 | 0.606896552 | 8 | 5 |
| MDGA2 | 0.88 | 3.473684211 | 0 | 0.75862069 | 8 | 4 |
| TENM4 | 1.76 | 3.473684211 | 0 | 0.75862069 | 8 | 4 |
| AS3MT | 1.76 | 2.315789474 | 0 | 0.75862069 | 8 | 4 |
| PCDHAC1 | 1.76 | 2.315789474 | 0 | 0.75862069 | 8 | 4 |
| P2RY8 | 1.32 | 2.315789474 | 2.2 | 1.137931034 | 9 | 8 |
| CSMD1 | 0.88 | 1.736842105 | 2.2 | 0.75862069 | 9 | 8 |
| UNC80 | 2.011428571 | 1.323308271 | 1.885714286 | 1.300492611 | 9 | 7 |
| DNAH5 | 1.173333333 | 1.543859649 | 1.466666667 | 1.011494253 | 9 | 6 |
| DSEL | 1.173333333 | 1.543859649 | 2.2 | 1.011494253 | 9 | 6 |
| NPAS2 | 1.173333333 | 1.543859649 | 2.2 | 1.011494253 | 9 | 6 |
| TAF1 | 1.173333333 | 1.543859649 | 2.2 | 1.011494253 | 9 | 6 |
| PLEKHA5 | 1.408 | 1.852631579 | 1.76 | 1.213793103 | 9 | 5 |
| GRIN2B | 1.408 | 1.852631579 | 1.76 | 0.606896552 | 9 | 5 |
| PRDM1 | 1.408 | 0.926315789 | 2.64 | 1.213793103 | 9 | 5 |
| SPHKAP | 0.704 | 0.926315789 | 2.64 | 0.606896552 | 9 | 5 |
| UNC5D | 1.76 | 1.157894737 | 2.2 | 1.517241379 | 9 | 4 |
| ARID5B | 0.88 | 1.157894737 | 2.2 | 0.75862069 | 9 | 4 |
| GFAP | 0.88 | 1.157894737 | 2.2 | 0.75862069 | 9 | 4 |
| PPFIA4 | 1.76 | 1.157894737 | 2.2 | 0.75862069 | 9 | 4 |
| SCN10A | 1.76 | 1.157894737 | 2.2 | 0.75862069 | 9 | 4 |
| ATP2A1 | 1.76 | 0 | 3.3 | 1.517241379 | 9 | 4 |
| PIM1 | 0.251428571 | 1.654135338 | 1.885714286 | 1.083743842 | 10 | 14 |
| DUSP2 | 0.541538462 | 2.137651822 | 1.353846154 | 1.400530504 | 10 | 13 |
| DST | 0.293333333 | 1.929824561 | 1.466666667 | 1.011494253 | 10 | 12 |
| ZNF804B | 0 | 2.315789474 | 1.1 | 1.517241379 | 10 | 8 |
| PRDM15 | 0 | 2.315789474 | 2.2 | 0.75862069 | 10 | 8 |
| CD79B | 0 | 1.157894737 | 1.65 | 0.75862069 | 10 | 8 |
| OSBPL10 | 0.502857143 | 1.323308271 | 1.885714286 | 1.300492611 | 10 | 7 |
| SPEF2 | 0 | 2.315789474 | 1.466666667 | 1.011494253 | 10 | 6 |
| BAZ2B | 0 | 1.852631579 | 1.76 | 1.820689655 | 10 | 5 |
| NRP1 | 0.704 | 1.852631579 | 1.76 | 1.213793103 | 10 | 5 |
| CLTCL1 | 0 | 2.315789474 | 1.1 | 1.517241379 | 10 | 4 |
| CSRNP3 | 0 | 2.315789474 | 2.2 | 1.517241379 | 10 | 4 |
| CSMD3 | 1.083076923 | 2.137651822 | 1.015384615 | 1.633952255 | 11 | 13 |
| GNA13 | 1.466666667 | 3.087719298 | 0.366666667 | 1.770114943 | 11 | 12 |
| PCNT | 1.173333333 | 1.929824561 | 0 | 1.517241379 | 11 | 12 |
| SGK1 | 0.96 | 2.105263158 | 0.4 | 2.206896552 | 11 | 11 |
| PKHD1L1 | 1.056 | 2.315789474 | 0.44 | 1.820689655 | 11 | 10 |
| AR | 1.408 | 1.852631579 | 0.88 | 1.517241379 | 11 | 10 |
| SDK1 | 0.782222222 | 2.058479532 | 0.488888889 | 2.022988506 | 11 | 9 |
| CNTNAP5 | 1.76 | 2.315789474 | 1.65 | 1.896551724 | 11 | 8 |
| IBTK | 0.88 | 2.315789474 | 0.55 | 1.517241379 | 11 | 8 |
| ZFP36L1 | 0.88 | 1.736842105 | 0 | 1.896551724 | 11 | 8 |
| TNFAIP3 | 1.32 | 1.736842105 | 0.55 | 1.517241379 | 11 | 8 |
| COL4A3 | 1.508571429 | 2.646616541 | 1.257142857 | 1.733990148 | 11 | 7 |
| LILRA1 | 1.005714286 | 1.984962406 | 0.628571429 | 1.733990148 | 11 | 7 |
| PIEZO2 | 1.005714286 | 1.984962406 | 0.628571429 | 1.733990148 | 11 | 7 |
| CENPF | 1.508571429 | 1.323308271 | 0.628571429 | 1.733990148 | 11 | 7 |
| ASXL3 | 1.408 | 2.778947368 | 0.88 | 1.820689655 | 11 | 5 |
| ANKS1B | 1.408 | 1.852631579 | 0.88 | 1.820689655 | 11 | 5 |
| SCN3A | 1.408 | 1.852631579 | 0.88 | 1.820689655 | 11 | 5 |
| SPTBN4 | 1.408 | 1.852631579 | 0.88 | 1.820689655 | 11 | 5 |
| NOS1 | 0.88 | 2.315789474 | 1.1 | 1.517241379 | 11 | 4 |
| SHANK2 | 0.88 | 2.315789474 | 0 | 1.517241379 | 11 | 4 |
| TP53 | 3.52 | 0.926315789 | 0.704 | 1.456551724 | 12 | 25 |
| SPEN | 1.955555556 | 1.543859649 | 0.977777778 | 1.348659004 | 12 | 9 |
| ANK2 | 2.2 | 2.315789474 | 1.1 | 1.517241379 | 12 | 8 |
| DYNC2H1 | 1.76 | 1.736842105 | 0 | 1.517241379 | 12 | 8 |
| PRKDC | 2.514285714 | 1.984962406 | 0.628571429 | 1.733990148 | 12 | 7 |
| FILIP1 | 2.011428571 | 1.984962406 | 1.257142857 | 1.300492611 | 12 | 7 |
| PKHD1 | 1.76 | 2.315789474 | 0 | 1.517241379 | 12 | 6 |
| LRRC7 | 2.112 | 2.778947368 | 0 | 1.820689655 | 12 | 5 |
| CDH9 | 2.112 | 2.778947368 | 0 | 1.213793103 | 12 | 5 |
| ZNF721 | 2.112 | 2.778947368 | 0 | 1.213793103 | 12 | 5 |
| DENND5B | 2.64 | 2.315789474 | 0 | 1.517241379 | 12 | 4 |
| OSBPL6 | 1.76 | 2.315789474 | 0 | 1.517241379 | 12 | 4 |
| TRPM6 | 2.64 | 1.157894737 | 1.1 | 1.517241379 | 12 | 4 |
| MYD88 | 0.704 | 0.231578947 | 4.4 | 1.062068966 | 13 | 20 |
| BCOR | 0 | 0 | 2.514285714 | 0.866995074 | 13 | 7 |
| TEX11 | 0 | 0.771929825 | 2.933333333 | 1.011494253 | 13 | 6 |
| COL4A5 | 0 | 0.926315789 | 3.52 | 1.213793103 | 13 | 5 |
| PAPPA2 | 0.704 | 0.926315789 | 3.52 | 1.213793103 | 13 | 5 |
| MYO1A | 0.704 | 0 | 2.64 | 1.213793103 | 13 | 5 |
| CCDC148 | 0 | 0 | 3.52 | 0.606896552 | 13 | 5 |
| RIOK2 | 0 | 0 | 3.3 | 1.517241379 | 13 | 4 |
| ITGB4 | 0 | 0 | 2.2 | 0.75862069 | 13 | 4 |
| KCNH7 | 0 | 0 | 4.4 | 0.75862069 | 13 | 4 |
| HLA-A | 0.704 | 1.235087719 | 2.346666667 | 1.618390805 | 14 | 15 |
| UBR4 | 0.812307692 | 0.712550607 | 1.692307692 | 1.400530504 | 14 | 13 |
| SETD1B | 0.586666667 | 0.385964912 | 1.466666667 | 1.517241379 | 14 | 12 |
| SI | 0.32 | 0.842105263 | 1.6 | 1.655172414 | 14 | 11 |
| USH2A | 0.704 | 0.463157895 | 1.76 | 1.820689655 | 14 | 10 |
| CSAG1 | 1.173333333 | 0.514619883 | 1.466666667 | 1.685823755 | 14 | 9 |
| MPDZ | 0 | 0.578947368 | 1.1 | 1.517241379 | 14 | 8 |
| SASH1 | 0.586666667 | 1.543859649 | 2.2 | 2.022988506 | 14 | 6 |
| NAV2 | 0.704 | 0.926315789 | 1.76 | 1.820689655 | 14 | 5 |
| NRAP | 0.704 | 0.926315789 | 1.76 | 1.820689655 | 14 | 5 |
| ADAMTS9 | 0.704 | 0.926315789 | 1.76 | 1.213793103 | 14 | 5 |
| ACSM2A | 0 | 0 | 2.64 | 1.820689655 | 14 | 5 |
| COL21A1 | 0 | 1.157894737 | 2.2 | 1.517241379 | 14 | 4 |
| RTL1 | 0.88 | 1.157894737 | 2.2 | 1.517241379 | 14 | 4 |
| DDHD1 | 0.88 | 0 | 2.2 | 2.275862069 | 14 | 4 |
| SYCE1 | 0 | 0 | 2.2 | 1.517241379 | 14 | 4 |
| TTN | 0.724705882 | 1.362229102 | 0.905882353 | 1.338742394 | 15 | 34 |
| DMD | 1.111578947 | 0.975069252 | 0.926315789 | 1.597096189 | 15 | 19 |
| SYNE2 | 0.621176471 | 1.907120743 | 1.294117647 | 1.427991886 | 15 | 17 |
| GPRIN1 | 1.242352941 | 1.634674923 | 1.294117647 | 1.606490872 | 15 | 17 |
| SDHA | 1.242352941 | 1.362229102 | 1.294117647 | 1.784989858 | 15 | 17 |
| NACAD | 0.88 | 1.447368421 | 1.65 | 1.706896552 | 15 | 16 |
| HLA-DRB1 | 1.32 | 1.447368421 | 0.825 | 1.517241379 | 15 | 16 |
| ZNF141 | 1.32 | 1.157894737 | 1.1 | 1.517241379 | 15 | 16 |
| DNAH9 | 1.005714286 | 1.984962406 | 1.571428571 | 1.517241379 | 15 | 14 |
| ATXN1 | 0.812307692 | 1.781376518 | 1.015384615 | 1.400530504 | 15 | 13 |
| RYR2 | 0.88 | 1.543859649 | 0.733333333 | 1.264367816 | 15 | 12 |
| ANKRD11 | 1.173333333 | 1.157894737 | 1.466666667 | 1.770114943 | 15 | 12 |
| LRP2 | 0.32 | 1.684210526 | 0.8 | 1.655172414 | 15 | 11 |
| COBL | 0.352 | 2.315789474 | 1.32 | 1.820689655 | 15 | 10 |
| ARAP2 | 0.88 | 1.736842105 | 1.1 | 1.896551724 | 15 | 8 |
| LOXHD1 | 0.88 | 1.736842105 | 1.1 | 1.896551724 | 15 | 8 |
| COL7A1 | 1.32 | 1.736842105 | 1.1 | 1.517241379 | 15 | 8 |
| MYCBP2 | 0.44 | 1.157894737 | 1.1 | 1.896551724 | 15 | 8 |
| APOB | 0.502857143 | 1.984962406 | 1.257142857 | 1.733990148 | 15 | 7 |
| BTG1 | 1.173333333 | 1.543859649 | 1.466666667 | 1.517241379 | 15 | 6 |
| TNRC6B | 1.173333333 | 1.543859649 | 1.466666667 | 1.517241379 | 15 | 6 |
| ROBO2 | 1.173333333 | 0.771929825 | 1.466666667 | 2.022988506 | 15 | 6 |
| UBE2A | 0.704 | 1.852631579 | 0.88 | 1.820689655 | 15 | 5 |
| FAM83B | 0.88 | 1.157894737 | 1.1 | 1.517241379 | 15 | 4 |
| KMT2D | 1.456551724 | 1.117967332 | 1.062068966 | 3.034483 | 16 | 29 |
| BCL2 | 1.173333333 | 1.984962406 | 0.628571429 | 2.600985222 | 16 | 21 |
| CREBBP | 1.173333333 | 1.102756892 | 0.628571429 | 2.311986864 | 16 | 21 |
| HLA-DQB1 | 1.54 | 1.447368421 | 0.825 | 2.086206897 | 16 | 16 |
| CACNA1A | 2.346666667 | 0.514619883 | 0.977777778 | 2.022988506 | 16 | 9 |
| EZH2 | 0.88 | 1.736842105 | 0.55 | 2.275862069 | 16 | 8 |
| ZAN | 1.005714286 | 1.323308271 | 0.628571429 | 2.167487685 | 16 | 7 |
| CARD11 | 0.502857143 | 0.661654135 | 0.628571429 | 2.167487685 | 16 | 7 |
| LAMA1 | 1.173333333 | 1.543859649 | 0 | 2.022988506 | 16 | 6 |
| TOP2A | 1.408 | 0.926315789 | 0 | 1.820689655 | 16 | 5 |
| STAT3 | 2.816 | 0 | 0 | 2.427586207 | 16 | 5 |

^*Num：Number of occurrences in 92 observed data sets.^

^The lightness of the color indicates the frequency of gene mutations in the data set, and the darker color represents the higher frequency.^

**Table S2.** The distribution of common mutations in each subgroup.

| **Clinical info** | **JAK-STAT** | **P-value*** | **BCL-CREBBP** | **P-value** | **MCD** | **P-value** | **TP53** | **P-value** | **Sparse itemset** | **P-value** |
| --- | --- | --- | --- | --- | --- | --- | --- | --- | --- | --- |
| **TP53** |  |  |  |  |  |  |  |  |  |  |
| **Mut** | **4(36.4%)** | **0.76** | **9(40.9%)** | **1.00** | **13(41.9%)** | **1.00** | **40(100.0%)** | **0.00** | **0(0.0%)** | **0.00** |
| **Wt** | **7(63.6%)** |  | **13(59.1%)** |  | **18(58.1%)** |  | **0(0.0%)** |  | **25(100.0%)** |  |
| **KMT2D** |  |  |  |  |  |  |  |  |  |  |
| **Mut** | **3(27.3%)** | **1.00** | **10(45.5%)** | **0.05** | **8(25.88%)** | **1.00** | **14(35.0%)** | **0.21** | **4(16.0%)** | **0.23** |
| **Wt** | **8(72.7%)** |  | **12(54.5%)** |  | **23(74.2%)** |  | **26(65.0%)** |  | **21(84.0%)** |  |
| **PIM1** | | | |  |  |  |  |  |  |  |
| **Mut** | **1(20.0%)** | **0.17** | **8(36.4%)** | **0.49** | **12(38.7%)** | **0.18** | **7(17.5%)** | **0.08** | **7(28.0%)** | **1.00** |
| **Wt** | **10(80.0%)** |  | **14(63.6%)** |  | **19(61.3%)** |  | **33(82.5%)** |  | **18(72.0%)** |  |
| **MYD88** | | | |  |  |  |  |  |  |  |
| **Mut** | **0(0.0%)** | **0.11** | **2(9.1%)** | **0.14** | **20(64.5%)** | **0.00** | **11(27.5%)** | **0.27** | **0(0.0%)** | **0.00** |
| **Wt** | **11(100.0%)** |  | **20(90.%)** |  | **11(35.5%)** |  | **29(72.5%)** |  | **25(100.0%)** |  |
| **CREBBP** |  |  |  |  |  |  |  |  |  |  |
| **Mut** | **4(36.4%)** | **0.07** | **15(68.2%)** | **0.00** | **2(6.5%)** | **0.13** | **7(17.5%)** | **0.89** | **0(0.0%)** | **0.00** |
| **Wt** | **7(63.6%)** |  | **7(31.8%)** |  | **29(93.5%)** |  | **33(82.5%)** |  | **25(100.0%)** |  |
| **CD79B** | | | |  |  |  |  |  |  |  |
| **Mut** | **0(0.0%)** | **0.20** | **5(22.7%)** | **0.53** | **17(54.8%)** | **0.00** | **4(10.0%)** | **0.16** | **0(0.0%)** | **0.00** |
| **Wt** | **11(100.0%)** |  | **17(77.3%)** |  | **14(45.2%)** |  | **36(90.0%)** |  | **25(100.0%)** |  |
| **B2M** | | | |  |  |  |  |  |  |  |
| **Mut** | **3(27.3%)** | **0.39** | **4(18.2%)** | **0.76** | **5(16.1%)** | **1.00** | **6(15.0%)** | **0.79** | **4(16.0%)** | **1.00** |
| **Wt** | **8(72.7%)** |  | **18(81.8%)** |  | **26(83.9%)** |  | **34(85.0%)** |  | **21(84.0%)** |  |
| **CCND3** | | | |  |  |  |  |  |  |  |
| **Mut** | **1(9.1%)** | **1.00** | **3(13.6%)** | **1.00** | **3(9.7%)** | **0.58** | **3(7.5%)** | **0.17** | **8(32.0%)** | **0.00** |
| **Wt** | **10(90.9%)** |  | **19(86.4%)** |  | **28(90.3%)** |  | **37(92.5%)** |  | **17(68.0%)** |  |
| **BCL2** | | | |  |  |  |  |  |  |  |
| **Mut** | **2(18.2%)** | **0.32** | **10(45.5%)** | **0.00** | **4(12.9%)** | **0.72** | **2(5.0%)** | **0.19** | **0(0.0%)** | **0.06** |
| **Wt** | **9(81.8%)** |  | **12(54.5%)** |  | **27(87.1%)** |  | **38(95.0%)** |  | **25(100.0%)** |  |
| **PCLO** | | | |  |  |  |  |  |  |  |
| **Mut** | **2(18.2%)** | **0.32** | **3(13.6%)** | **0.69** | **2(6.5%)** | **0.49** | **3(7.5%)** | **0.51** | **3(12.0%)** | **0.72** |
| **Wt** | **9(81.8%)** |  | **19(86.4%)** |  | **29(93.5%)** |  | **37(92.5%)** |  | **22(88.0%)** |  |
| **TET2** | | | |  |  |  |  |  |  |  |
| **Mut** | **1(9.1%)** | **1.00** | **4(18.2%)** | **0.46** | **5(16.1%)** | **0.52** | **3(7.5%)** | **0.38** | **3(12.0%)** | **1.00** |
| **Wt** | **10(90.9%)** |  | **18(81.8%)** |  | **26(83.9%)** |  | **37(92.5%)** |  | **22(88.0%)** |  |
| **TNFAIP3** | | | |  |  |  |  |  |  |  |
| **Mut** | **2(18.2%)** | **1.00** | **0(0.0%)** | **0.06** | **1(3.2%)** | **0.10** | **4(10.0%)** | **0.75** | **6(24.0%)** | **0.07** |
| **Wt** | **9(81.8%)** |  | **22(100.0%)** |  | **30(96.7%)** |  | **36(90.0%)** |  | **19(76.0%)** |  |
| **CARD11** | | | |  |  |  |  |  |  |  |
| **Mut** | **2(18.2%)** | **0.61** | **1(4.5%)** | **0.44** | **5(16.1%)** | **0.33** | **4(10.0%)** | **0.76** | **3(12.0%)** | **1.00** |
| **Wt** | **9(81.8%)** |  | **21(95.5%)** |  | **26(83.9%)** |  | **36(90.0%)** |  | **22(88.0%)** |  |
| **DDX3X** | | | |  |  |  |  |  |  |  |
| **Mut** | **1(9.1%)** | **1.00** | **4(18.2%)** | **0.20** | **1(3.2%)** | **0.26** | **6(15.0%)** | **0.18** | **1(4.0%)** | **0.43** |
| **Wt** | **10(90.9%)** |  | **18(81.8%)** |  | **30(96.8%)** |  | **34(85.0%)** |  | **24(96.0%)** |  |
| **PRDM1** | | | |  |  |  |  |  |  |  |
| **Mut** | **0(0.0%)** | **0.59** | **0(0.0%)** | **0.11** | **4(12.9%)** | **0.46** | **3(7.5%)** | **0.27** | **4(16.0%)** | **0.23** |
| **Wt** | **11(100.0%)** |  | **22(100.0%)** |  | **27(87.1%)** |  | **37(92.5%)** |  | **21(84.0%)** |  |
| **EZH2** | | | |  |  |  |  |  |  |  |
| **Mut** | **1(9.1%)** | **0.59** | **4(18.2%)** | **0.05** | **2(6.5%)** | **1.00** | **4(10.0%)** | **0.45** | **0(0.0%)** | **0.18** |
| **Wt** | **10(90.9%)** |  | **18(81.8%)** |  | **29(93.5%)** |  | **36(90.0%)** |  | **25(100.0%)** |  |
| **MYC** | | | |  |  |  |  |  |  |  |
| **Mut** | **4(36.4%)** | **0.00** | **3(13.6%)** | **0.19** | **0(0.0%)** | **0.10** | **3(7.5%)** | **1.00** | **2(8.0%)** | **1.00** |
| **Wt** | **7(63.6%)** |  | **19(86.4%)** |  | **31(100.0%)** |  | **37(92.5%)** |  | **23(92.0%)** |  |
| **PTEN** | | | |  |  |  |  |  |  |  |
| **Mut** | **0(0.0%)** | **1.00** | **3(13.6%)** | **0.19** | **2(6.5%)** | **1.00** | **3(7.5%)** | **1.00** | **1(4.0%)** | **0.67** |
| **Wt** | **11(100.0%)** |  | **19(86.4%)** |  | **29(93.5%)** |  | **37(92.5%)** |  | **24(96.0%)** |  |
| **TBL1XR1** | | | |  |  |  |  |  |  |  |
| **Mut** | **2(18.2%)** | **0.18** | **2(9.1%)** | **0.66** | **2(6.5%)** | **1.00** | **1(2.5%)** | **0.23** | **1(4.0%)** | **0.67** |
| **Wt** | **9(81.8%)** |  | **20(90.9%)** |  | **29(93.5%)** |  | **39(97.5%)** |  | **24(96.0%)** |  |
| **TNFRSF14** | | | |  |  |  |  |  |  |  |
| **Mut** | **2(18.2%)** | **0.18** | **5(22.7%)** | **0.01** | **0(0.0%)** | **0.09** | **4(10.0%)** | **0.45** | **1(4.0%)** | **0.67** |
| **Wt** | **9(81.8%)** |  | **17(77.3%)** |  | **31(100.0%)** |  | **36(90.0%)** |  | **24(96.0%)** |  |
| **FAT4** | | | |  |  |  |  |  |  |  |
| **Mut** | **0(0.0%)** | **1.00** | **1(4.5%)** | **1.00** | **3(9.7%)** | **0.68** | **1(2.5%)** | **0.23** | **3(12.0%)** | **0.37** |
| **Wt** | **11(100.0%)** |  | **21(95.5%)** |  | **28(90.3%)** |  | **39(97.5%)** |  | **22(88.0%)** |  |
| **SOCS1** | | | |  |  |  |  |  |  |  |
| **Mut** | **8(72.7%)** | **0.00** | **3(13.6%)** | **0.38** | **0(0.0%)** | **0.05** | **2(5.0%)** | **0.68** | **0(0.0%)** | **0.11** |
| **Wt** | **3(27.3%)** |  | **19(86.4%)** |  | **31(100.0%)** |  | **38(95.0%)** |  | **25(100.0%)** |  |
| **EP300** | | | |  |  |  |  |  |  |  |
| **Mut** | **0(0.0%)** | **1.00** | **1(4.5%)** | **1.00** | **3(9.7%)** | **0.09** | **0(0.0%)** | **0.14** | **0(0.0%)** | **0.57** |
| **Wt** | **11(100.0%)** |  | **21(95.5%)** |  | **28(90.3%)** |  | **40(100.0%)** |  | **25(100.0%)** |  |
| **SPEN** | | | |  |  |  |  |  |  |  |
| **Mut** | **0(0.0%)** | **0.80** | **2(9.1%)** | **0.62** | **3(9.7%)** | **0.38** | **2(5.0%)** | **1.00** | **2(8.0%)** | **0.65** |
| **Wt** | **11(100.0%)** |  | **20(90.9%)** |  | **28(90.3%)** |  | **38(95.0%)** |  | **23(92.0%)** |  |
| **STAT6** | | | |  |  |  |  |  |  |  |
| **Mut** | **6(54.5%)** | **0.00** | **2(9.1%)** | **0.62** | **0(0.0%)** | **0.17** | **3(7.5%)** | **0.69** | **0(0.0%)** | **0.33** |
| **Wt** | **5(45.5%)** |  | **20(90.9%)** |  | **31(100.0%)** |  | **37(92.5%)** |  | **25(100.0%)** |  |
| **NOTCH1** | | | |  |  |  |  |  |  |  |
| **Mut** | **0(0.0%)** | **1.00** | **0(0.0%)** | **0.59** | **1(3.2%)** | **0.30** | **2(5.0%)** | **1.00** | **2(8.0%)** | **0.60** |
| **Wt** | **11(100.0%)** |  | **22(100.0%)** |  | **30(96.8%)** |  | **38(95.0%)** |  | **23(92.0%)** |  |
| **BCOR** | | | |  |  |  |  |  |  |  |
| **Mut** | **0(0.0%)** | **1.00** | **0(0.0%)** | **0.57** | **2(6.5%)** | **0.59** | **1(2.5%)** | **0.64** | **2(8.0%)** | **0.28** |
| **Wt** | **11(100.0%)** |  | **22(100.0%)** |  | **29(93.5%)** |  | **39(97.5%)** |  | **23(92.0%)** |  |
| **NOTCH2** | | | |  |  |  |  |  |  |  |
| **Mut** | **0(0.0%)** | **1.00** | **0(0.0%)** | **0.59** | **3(9.7%)** | **0.32** | **3(7.5%)** | **0.65** | **1(4.0%)** | **1.00** |
| **Wt** | **11(100.0%)** |  | **22(100.0%)** |  | **28(90.3%)** |  | **37(92.5%)** |  | **24(96.0%)** |  |
| **CD58** | | | |  |  |  |  |  |  |  |
| **Mut** | **2(18.2%)** | **0.03** | **1(4.5%)** | **0.55** | **1(3.2%)** | **1.00** | **2(5.0%)** | **0.57** | **0(0.0%)** | **0.57** |
| **Wt** | **9(81.8%)** |  | **21(95.5%)** |  | **30(96.7%)** |  | **38(95.0%)** |  | **25(100.0%)** |  |
| **GNA13** | | | |  |  |  |  |  |  |  |
| **Mut** | **0(0.0%)** | **1.00** | **0(0.0%)** | **1.00** | **1(3.2%)** | **1.00** | **2(5.0%)** | **0.57** | **1(4.0%)** | **1.00** |
| **Wt** | **11(100.0%)** |  | **22(100.0%)** |  | **30(96.7%)** |  | **38(95.0%)** |  | **24(96.0%)** |  |
| **ITPKB** | | | |  |  |  |  |  |  |  |
| **Mut** | **2(18.2%)** | **0.03** | **1(4.5%)** | **0.55** | **1(3.2%)** | **1.00** | **1(2.5%)** | **1.00** | **0(0.0%)** | **0.57** |
| **Wt** | **9(81.8%)** |  | **21(95.5%)** |  | **30(96.7%)** |  | **39(97.5%)** |  | **25(100.0%)** |  |
| **STAT3** | | | |  |  |  |  |  |  |  |
| **Mut** | **3(27.3%)** | **0.00** | **0(0.0%)** | **0.57** | **0(0.0%)** | **0.30** | **2(5.0%)** | **1.00** | **1(4.0%)** | **1.00** |
| **Wt** | **8(72.7%)** |  | **22(100.0%)** |  | **31(100.0%)** |  | **38(95.0%)** |  | **24(96.0%)** |  |

* P value: Chi-square test result of the comparison between the subgroup and the patients excluding the subgroup

**Table S3.** Summary of clinical information for each subgroup.

| **Clinical info** | **JAK-STAT** | **P-value*** | **BCL-CREBBP** | **P-value** | **MCD** | **P-value** | **TP53** | **P-value** | **Sparse itemset** | **P-value** |
| --- | --- | --- | --- | --- | --- | --- | --- | --- | --- | --- |
| **Age** |  |  |  |  |  |  |  |  |  |  |
| **≤60** | **6(66.7%)** | **1.00** | **13(61.9%)** | **0.60** | **14(51.9%)** | **0.05** | **26(72.2%)** | **0.49** | **17(73.9%)** | **0.45** |
| **＞60** | **3(33.3%)** |  | **8(38.1%)** |  | **13(48.1%)** |  | **10 (27.8%)** |  | **6(26.1%)** |  |
| **Gender** |  |  |  |  |  |  |  |  |  |  |
| **Male** | **4(44.4%)** | **0.73** | **9(42.9%)** | **0.45** | **14(51.9%)** | **1.00** | **21(58.3%)** | **0.23** | **11(47.8%)** | **0.80** |
| **Female** | **5(55.6%)** |  | **12(57.1%)** |  | **13(48.1%)** |  | **15(41.7%)** |  | **12(52.2%)** |  |
| **Pathological subtype (COO)** | | | |  |  |  |  |  |  |  |
| **GCB** | **6(66.7%)** | **0.06** | **14(66.7%)** | **0.00** | **5(18.5%)** | **0.05** | **14(38.9%)** | **0.49** | **3(13.0%)** | **0.02** |
| **Non-GCB** | **3(33.3%)** |  | **7(33.3%)** |  | **22(81.5%)** |  | **22(61.1%)** |  | **20(87.0%)** |  |
| **Ann arbor stage** | | | |  |  |  |  |  |  |  |
| **I** | **1(11.1%)** | **0.48** | **1(4.8%)** | **0.23** | **0(0.0%)** | **0.18** | **0(0.0%)** | **0.57** | **2(8.7%)** | **0.08** |
| **II** | **0(0.0%)** |  | **1(4.8%)** |  | **4(14.8%)** |  | **3(8.3%)** |  | **0(0.0%)** |  |
| **III** | **1(11.1%)** |  | **3(14.3%)** |  | **1(3.8%)** |  | **2(5.5%)** |  | **0(0.0%)** |  |
| **IV** | **7(77.8%)** |  | **16(76.2%)** |  | **22 (81.5%)** |  | **31(86.1%)** |  | **21(91.3%)** |  |
| **IPI** |  |  |  |  |  |  |  |  |  |  |
| **1-2** | **3(30.0%)** | **0.71** | **6(26.1%)** | **1.00** | **6(22.2%)** | **0.80** | **8(19.0%)** | **0.24** | **5(25.0%)** | **1.00** |
| **≥3** | **7(70.0%)** |  | **17(73.9%)** |  | **21(77.8%)** |  | **34(81.0%)** |  | **15(75.0%)** |  |
| **Performance status(ECOG)** | | | |  |  |  |  |  |  |  |
| **0-2** | **6(66.7%)** | **1.00** | **11(52.4%)** | **0.06** | **16(59.3%)** | **0.21** | **24(66.7%)** | **0.64** | **18(78.3%)** | **0.43** |
| **≥2** | **3(33.3%)** |  | **10(47.6%)** |  | **11(40.8%)** |  | **12(33.3%)** |  | **5(21.7%)** |  |
| **Induction therapy response** | | | |  |  |  |  |  |  |  |
| **CR** | **0(0.0%)** | **0.10** | **5(23.8%)** | **1.00** | **6(22.2%)** | **0.79** | **10(27.8%)** | **0.62** | **6(26.1%)** | **1.00** |
| **Non-CR** | **9(100.0%)** |  | **16(76.2%)** |  | **21(77.8%)** |  | **26(72.2%)** |  | **17(73.9%)** |  |
| **Salvage treatment response** | | | |  |  |  |  |  |  |  |
| **CR** | **1(11.1%)** | **1.00** | **2(9.5%)** | **0.50** | **4(14.8%)** | **1.00** | **3(8.3%)** | **0.22** | **6(26.1%)** | **0.10** |
| **Non-CR** | **8(88.9%)** |  | **19(90.4%)** |  | **23(85.2%)** |  | **33(91.7%)** |  | **17(73.9%)** |  |
| **CART treatment** | | | |  |  |  |  |  |  |  |
| **Yes** | **8(88.9%)** | **0.67** | **16(76.2%)** | **0.76** | **20(74.1%)** | **0.57** | **28(77.8%)** | **1.00** | **20(87.0%)** | **0.37** |
| **No** | **1(11.1%)** |  | **5(23.8%)** |  | **7(25.9%)** |  | **8(22.2%)** |  | **3(13.0%)** |  |
| **CART treatment response** | | | |  |  |  |  |  |  |  |
| **CR** | **3(37.5%)** | **1.00** | **5(31.3%)** | **0.56** | **6(30.0%)** | **0.42** | **6(21.4%)** | **0.02** | **11(55.0%)** | **0.10** |
| **Non-CR** | **5(62.5%)** |  | **11(68.7%)** |  | **14(70.0%)** |  | **22(78.6%)** |  | **9(45.0%)** |  |
| **Central Nervous System Lymphoma** | | | |  |  |  |  |  |  |  |
| **Yes** | **0(0.0%)** | **0.59** | **3(14.3%)** | **0.41** | **3(11.1%)** | **0.70** | **3(8.3%)** | **1.00** | **3(13.0%)** | **0.68** |
| **No** | **9(100.0%)** |  | **18(85.7%)** |  | **23(88.9%)** |  | **33(91.7%)** |  | **20(87.0%)** |  |

* P value: Chi-square test result of the comparison between the subgroup and the patients excluding the subgroup
