# Supplementary material for: Genetic Landscape of Relapsed and Refractory Diffuse Large B-Cell Lymphoma: A Systemic Review and Association Analysis With Next-Generation Sequencing
Source: Front Genet. 2021 Dec 2;12:677650. doi: 10.3389/fgene.2021.677650 (PMC8675234; doi:10.3389/fgene.2021.677650)
Supplement: Supplementary file 2 [file DataSheet1.docx]

Supplementary Material 1

# Supplementary Method

## Methods for standardizing mutation type labels in 4 studies

The methods for standardizing mutation type labels are as follows:

(1) Splice site SNV: Splice site SNV, sSp, splice_acceptor_variant, splice_donor_variant, splice_region_variant.

(2) Truncating SNV: Truncating SNV, nonsense, stop_gained.

(3) Indel: Deletion, Insertion, FS Del, nonFS Del, FS Ins, iFS, il-, il+, iFS, frameshift_variant, transcript_ablation, disruptive_inframe_deletion, start_lost, exon_loss_variant.

(4) Missense SNV: Missense SNV, missense, sS-, sms, missense_variant, stop_lost, stop_retained_variant, initiator_codon_variant, 5_prime_UTR_premature_start_codon_gain_variant.

(5) Synonymous SNV: Synonymous SNV.

## Apriori algorithm

For more information on Supplementary Material and for details on the different file types accepted, pleas Apriori is the core algorithm in association analysis and its core ideas are as follows：let I = {i1,i2,...,in} be a set of n binary attributes called items and let D = {t1,t2,...,tm} be a set of transactions called the database; each transaction in D has a unique transaction ID and contains a subset of the items in I. A rule is deﬁned as an implication of the form X ⇒ Y, where X,Y ⊆ I and X ∩Y = ∅. The support supp(X) of an itemset X is deﬁned as the proportion of transactions in the data set that contain the itemset. The confidence of a rule is defined as conf(X ⇒ Y ) = supp(X ∪Y )/supp(X). The lift of a rule is defined as lift(X ⇒ Y ) = supp(X ∪Y )/(supp(X)supp(Y ).

In our study, I is a collection of gene mutation types and D is a collection of individual patients. Based on the above algorithm, we analyzed the association of gene mutations in RRDLBCL patients. Our calculation process is based on the R package *arules* (Version 1.6-4) [^[[1]](#footnote-1)^]. The minimum support for screening frequent itemsets is 0.03 and the minimum confidence is 0.1. At the same time, the lift value of rules is counted. A lift associated with two genes that is < 1 indicates a lack of correlation between the two.

## Self- and Super-organizing Maps (SOMs) Realization and visualization of genetic classification of 92 patients

SOMs have seen many diverse applications in a broad range of fields, including medicine, biology, chemistry, image analysis, speech recognition, engineering, computer science, and many more. We used the R-based kohonen package to implement this algorithm^[[2]](#footnote-2)^. The SOM consists m x n grid of output nodes, which are connected to each input vector. Each output node has the same dimensionality as the input vectors but is initialized with random data. The input data are mapped onto the SOM based on the Euclidian distance between the input vector and the best matching output node. Similar input vectors are then grouped together based on a neighborhood function.

We apply this algorithm to the selected hot genes, using the lift value (derived by the apriori algorithm) for a major gene mutation (such as SOCS1) as a variable. There are six genes used to provide lift values as variables: SOCS1, MYD88, KMT2D and TP53. The weight planes of the SOM were used to visualize the non-linear relationship between the variables via the color gradients of the z-score color scale. A k-means algorithm was used to create clusters of similar groups of input vectors on the SOM. Supervised mapping, where a dependent variable (categorical or continuous) is available, is implemented in the *xyf* function of the kohonen package. We applied this supervised algorithm to analyze the ideal degree of clustering.

## Methods of analysis based on RNA-seq and GO analysis

The linear correlation between gene expressions was analyzed using the R language (*cor.test* function), and the correlation matrix used the *cor* function to obtain the Pearson correlation coefficient results and used the pheatmap package to plot. Go analysis was done with clusterProfiler package (*enrichGO* function, v4.0.2). Based on the R language survival package (version 4.1.0).

## Methods of analysis based on RNA-seq and GO analysis Visualization of genetic classification of 92 patients

Based on the analysis of this study, we believe that there may be several combinations in relapsed and refractory diffuse large B-cell lymphoma, which can summarize most of the mutation types. We classify observations based on certain rules, and visualization is achieved through the *pheatmap* package^[[3]](#footnote-3)^. The specific rules are as follows.

(1) JAK-STAT type. Including at least one major mutations of SOCS1 or STAT6;

(2) BCL2-CREBBP type. Including at least one major mutations of BCL2 and CREBBP.

(3) MCD type. Including at least one major mutations of MYD88 and CD79B.

(4) TP53 mutation. And TP53 independent refers to TP53 mutation without co-mutation as SOCS1, STAT6, BCL2, CREBBP, MYD88 and CD79B.

(5) Undefined (Sparse-item). Patients without mutations of SOCS1, STAT6, BCL2, CREBBP, MYD88, CD79B and TP53.

In addition, patients with JAK-STAT type, BCL-CREBBP type or MCD type at the same time are marked as Complex types in the heat map.

## Methods of targeted sequencing

A core panel of 339 genes selected on the basis of prior implication in the pathogenesis of hematologic disease was analyzed in R&R DLBCL disease. The complete set of biotinylated long oligonucleotide probes provided by Roche NimbleGen to perform sequence capture of 339 genes (all coding exons). Two-hundred twenty-five nanograms of genomic DNA extracted from frozen tumor tissues were used to generate DNA libraries. Five 10 µm-thick sections per sample were used to extract DNA using the QIAamp DNA Mini Kit according to the manufacturer's instructions (Qiagen). The probe pool was hybridized to 250ng of gDNA upstream and downstream of each region of interest. Pooled DNA libraries were loaded onto the cBot System for cluster generation followed by 2×150 paired-end sequencing on NextSeq550 sequencer (both from Illumina). Average depth of coverage across the targeted regions was ~3000. Paired‐end reads which is in FASTQ format were mapped to the human genome (NCBI build 37) using BWA control software with default parameters. Mutational signatures were called on the final VCF file by Vardict and MuTect2 software in terms of the BAM file which is the output of the previous analysis.

Based on the R language survival package (version 4.1.0), Log-Rank test is used for survival analysis and visualization; Chi-square test and Fisher's exact test are used to evaluate the comparison of clinical information of different subtypes, both based on R package. The method of association analysis and application is the same as the published data.

1. Borgelt C (2004). Finding Association Rules/Hyperedges with the Apriori Algorithm. Working Group Neural Networks and Fuzzy Systems, Otto-von-Guericke-University of Magdeburg, Universit¨atsplatz 2, D-39106 Magdeburg, Germany. URL https://cran.r-project.org/web/packages/arules/arules.pdf [↑](#footnote-ref-1)
2. Wehrens R, Kruisselbrink J (2018). kohonen: Supervised and Unsupervised Self-Organising Maps. R package version 3.0.7, URL https://CRAN.R-project.org/package=kohonen. [↑](#footnote-ref-2)
3. https://cran.r-project.org/web/packages/pheatmap/pheatmap.pdf [↑](#footnote-ref-3)
